# Identification of a Circular RNA as a Potential Diagnostic and Prognostic Biomarker in Breast Cancer Through Integrated Bioinformatic and Experimental Analyses

**DOI:** 10.1002/ansa.70098

**Published:** 2026-07-28

**Authors:** MohamadReza Hashemi, Pejman Morovat, Azin Khoshghiafeh, Novin Nikbakhsh, Mohamadreza Ahmadifard

**Affiliations:** ^1^ Cellular and Molecular Biology Research Center, Health Research Institute Babol University of Medical Sciences Babol Iran; ^2^ Department of Medical Genetics Faculty of Medicine Babol University of Medical Sciences Babol Iran; ^3^ Department of Medical Biotechnology, Faculty of Medicine Babol University of Medical Sciences Babol Iran; ^4^ Department of Surgery, Faculty of Medicine Babol University of Medical Sciences Babol Iran

**Keywords:** biomarkers, breast neoplasms, circular RNA, competitive endogenous RNA, cyclin B1

## Abstract

Breast cancer (BC), which has complex molecular subgroups that contribute to a range of clinical outcomes, is still one of the top causes of morbidity and death among women globally. This challenge necessitates ongoing research to improve early detection, treatment strategies, and patient outcomes. Recent research has shown that circular RNAs (circRNAs) influence gene expression through various mechanisms, particularly by functioning as competing endogenous RNAs (ceRNAs). Moreover, numerous circRNAs have been implicated in the initiation and progression of various tumor types. Yet, the expression and functional roles of multiple circRNAs remain largely unexplored in specific cancers. In this study, circRNA expression data from three Gene Expression Omnibus datasets were analyzed to identify candidate circRNAs associated with BC. Reverse‐transcription quantitative polymerase chain reaction (RT‐qPCR) was performed to validate the candidate circRNAs in tissue samples from 24 BC patients. Using bioinformatic analyses, downstream target microRNAs and messenger RNAs of these circRNAs were probed for the construction of a ceRNA regulatory network. A combined number of 40 distinct circRNAs with differential expression were identified, among which hsa_circ_0000231 and hsa_circ_0011385 were selected based on their novelty and potential to function as ceRNAs, and a corresponding ceRNA network was built. RT‐qPCR revealed that hsa_circ_0000231 is significantly upregulated in BC tissues compared to adjacent non‐cancerous tissues. Additionally, based on bioinformatic investigations, hsa_circ_0000231/hsa‐miR‐5683/cyclin B1 regulatory axis was predicted. By combining cross‐dataset computational screening with targeted experimental validation and diagnostic accuracy assessment, this study presents a systematic framework for translating high‐throughput RNA data into quantifiable biomarker candidates with potential clinical relevance. These findings may contribute to the identification of a novel diagnostic and prognostic biomarker in BC.

## Graphical abstract

Forty circular RNAs were found to be significantly dysregulated in microarray datasets. Expression levels of hsa_circ_0000231 were significantly upregulated in breast cancer (BC) tissues, while hsa_circ_0011385 exhibited no significant difference in expression levels. This study also revealed that hsa_circ_0000231/hsa‐miR‐5683/CCNB1 regulatory axis may contribute to BC tumorigenesis.

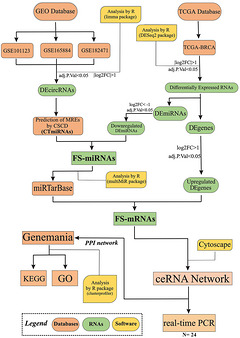

**Abbreviations**
AS: atherosclerosis
AUC: area under the curve
BC: breast cancer
BP: biological process
BRCA: breast invasive carcinoma
CC: cellular component
CCNB1: cyclin B1
ceRNA: competing endogenous RNA
circRNAs: circular RNAs
CRC: colorectal cancer
CSCD: Cancer‐Specific CircRNA Database
DEcircRNAs: differentially expressed circRNAs
DEgenes: differentially expressed messenger RNAs
DEmiRNAs: differentially expressed microRNAs
FmRNAs: final messenger RNAs
FS‐miRNAs: final shared microRNAs
FS‐mRNAs: final shared messenger RNAs
GEO: Gene Expression Omnibus
GO: Gene Ontology
KEGG: Kyoto Encyclopedia of Genes and Genomes
MF: molecular function
miRNAs: microRNAs
MREs: microRNA response elements
NCBI: National Center for Biotechnology Information
ncRNAs: noncoding RNAs
OC: ovarian cancer
PCA: principal component analysis
PDAC: pancreatic ductal adenocarcinoma
PPI: protein‐protein interaction
ROC: receiver operating characteristic
RT‐qPCR: reverse‐transcription quantitative polymerase chain reaction
TCGA: The Cancer Genome Atlas

## 1 Introduction

Breast cancer (BC) ranks among the most prevalent malignancies and is the primary cause of cancer‐related mortality among women globally [1]. With multiple recognized histotypes and molecular subtypes, it is a biologically and clinically heterogeneous disease with varying etiologies, risk factor profiles, responses to treatment, and prognoses [2]. BC patients who do not have metastases show a 5‐year overall survival rate of over 80%; however, distant metastases can severely reduce this rate to almost 25% [3]. Although mammography has significantly improved BC detection over recent decades, limitations such as false negative results and the potential risk of radiation‐induced malignancy have contributed to tissue biopsy followed by histological examination remaining the gold standard for BC diagnosis [4]. This often results in the diagnosis of BC at more advanced stages, thereby increasing the risk of metastasis and reducing overall survival. Therefore, advancements in molecular investigations contribute to a deeper understanding of pathways underlying breast tumor growth and may facilitate the identification of diagnostic biomarkers and the development of innovative treatment approaches.

Emerging evidence highlights noncoding RNAs (ncRNAs) as key epigenetic regulators that serve essential functions in several aspects of cancer biology. Circular RNAs (circRNAs) are a unique class of ncRNAs that have gained increasing attention in recent years due to their distinct characteristics and involvement in diverse biological processes (BPs) [5]. circRNAs are covalently closed loops formed through back‐splicing. Due to the absence of a 5’ cap and 3’ poly‐A tail, circRNAs exhibit enhanced resistance to RNases and exonucleases, rendering them more stable than linear RNAs [5, 6]. Recent studies have highlighted the exceptional functional diversity of circRNAs, which are mediated through their interactions with various molecular targets, including RNA, DNA, and proteins [7]. CircRNAs’ ability to function as competing endogenous RNA (ceRNA) has been studied considerably in contemporary research. According to the ceRNA hypothesis [8], circRNAs can sequester microRNAs (miRNAs) and hamper their binding to target messenger RNAs (mRNAs), thus preventing mRNA degradation and regulating gene expression at the post‐transcriptional level. The abnormal expression of circRNAs has been extensively documented across various cancer types, with a notable emphasis on BC [7]. They have been shown to impact different mechanisms involved in the initiation and progression of BC, including proliferation, apoptosis, and metastasis, functioning either as oncogenes or tumor suppressors [9, 10]. For example, Yao et al. demonstrated that circFAT1 acts as an oncogene in BC via sponging of miR‐525‐5p, subsequently regulating SKA1 expression [11]. A study by Ding et al. indicated that circWHSC1 plays a tumor suppressive role in BC through sequestering miR‐212‐5p to regulate the expression of AKT3 [12]. Despite advancements in high‐throughput technologies, such as RNA sequencing, that have enabled the discovery of numerous circRNAs, their precise regulatory mechanisms in BC have not been fully understood.

Despite the growing availability of high‐throughput RNA data, the identification of clinically relevant circRNA biomarkers remains challenged by inconsistent selection criteria and limited experimental validation. To address these limitations, there is a need for structured analytical strategies that integrate data processing, differential expression analysis, candidate prioritization, and independent validation within a unified workflow. In this context, the present study implements a multi‐step approach that combines cross‐dataset bioinformatic screening with functional analysis and experimental verification, aiming to improve the robustness, reproducibility, and interpretability of circRNA biomarker discovery in BC.

**FIGURE 1:**
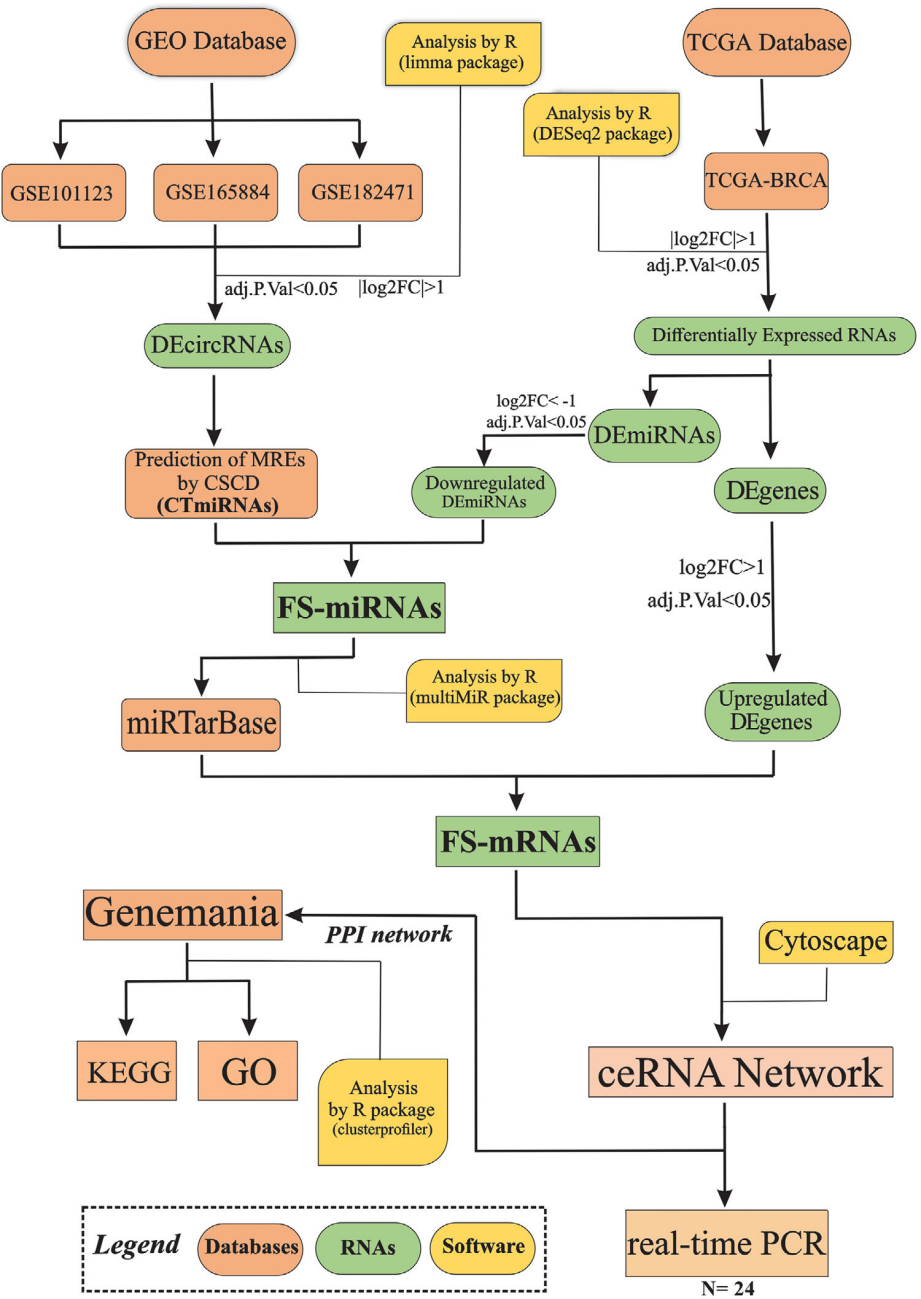
Overview of the analytical workflow for breast cancer expression data. Differential expression analysis of circular RNAs (circRNAs), miRNAs, and mRNAs was performed using GEO (GSE101123, GSE165884, and GSE182471) and The Cancer Genome Atlas‐breast invasive carcinoma (TCGA‐BRCA) data. MicroRNA response elements (MREs) were predicted via Cancer‐Specific CircRNA Database (CSCD). MiRNA‐mRNA interactions were retrieved from miRTarBase and multiMiR. The expression of selected circRNAs was experimentally validated in clinical tissue samples using real‐time polymerase chain reaction (PCR). Subsequently, protein‐protein interactions (PPIs) were analyzed utilizing GeneMANIA and Cytoscape. Finally, Pathway and functional enrichment analysis were conducted using Kyoto Encyclopedia of Genes and Genomes (KEGG) and Gene Ontology (GO).

We used the National Center for Biotechnology Information Gene Expression Omnibus (NCBI GEO) [13] to identify BC‐related circRNAs. Among the 40 identified circRNAs, hsa_circ_0000231 and hsa_circ_0011385 were prioritized for further study based on their consistent upregulation across datasets, their novelty in BC (with no prior tissue‐based validation), and their predicted ability to function as ceRNAs. These features made them promising candidates for subsequent experimental validation. The expression of hsa_circ_0000231 was subsequently validated using reverse‐transcription quantitative polymerase chain reaction (RT‐qPCR) as a potential biomarker. Furthermore, by analyzing miRNA and mRNA sequencing data from The Cancer Genome Atlas (TCGA) [14] and applying complementary bioinformatic tools, we proposed hsa_circ_0000231/hsa‐miR‐5683/CCNB1 as a potential regulatory axis for further investigation. The flowchart of this study is illustrated in Figure 1.

## 2 Materials and Methods

### 2.1 Identification of Differentially Expressed RNAs

Primarily, BC‐related circRNA expression data were obtained from GEO (http://www.ncbi.nlm.nih.gov/geo/) as a functional genomic database for identification of differentially expressed circRNAs (DEcircRNAs), by utilizing the GEOquery package (http://www.bioconductor.org/packages/release/bioc/html/GEOquery.html) in R software (V 4.2.2). Three microarray datasets were used in this study: GSE101123 (including eight BC tissues and three normal mammary gland tissues), GSE165884 (including four BC tissues and four adjacent non‐tumor tissues), and GSE182471 (including five BC tissues and five adjacent non‐tumor tissues). GSE165884 and GSE182471 datasets both used gene chips with the same platform (074301 Arraystar Human CircRNA microarray V2), while the GSE101123 dataset employed gene chips based on a different platform (Agilent‐069978 Arraystar Human CircRNA microarray V1). Furthermore, R software's sva package (V 3.46.0) [15] was utilized to merge three datasets and remove the hidden batch effects. To correct for potential batch effects caused by differences in experimental platforms and processing, we applied the ComBat function from the sva package. This method uses an empirical Bayes framework to adjust expression values by modeling and removing batch‐specific means and variances, while retaining true biological variability. After batch correction, we performed principal component analysis (PCA) to confirm that the samples grouped according to biological condition rather than dataset origin. DEcircRNAs were identified by using the Limma Bioconductor package (V 3.54.0) [16], and the criteria of |log2FoldChange| > 1 and adjusted *p*‐value < 0.05 were considered statistically significant.

The miRNA‐Seq and RNA‐Seq expression data of breast invasive carcinoma (BRCA) were extracted from TCGA on 22 February, 2023. The miRNA sequencing data are composed of 1078 primary tumor samples and 104 normal controls, whereas RNA sequencing data are comprised of 1091 primary tumor samples and 113 normal controls. The R package GDCRNATools [17] was applied with TMM [18] and VOOM [19] approaches for normalization of these data. Additionally, the Bioconductor package DESeq2 (V 1.38.0) was used for differential expression analysis of miRNA‐Seq and RNA‐Seq expression data. Screening of differentially expressed miRNAs (DEmiRNAs) and differentially expressed mRNAs (DEgenes) was performed based on the criteria of |log2FoldChange| > 1 and adjusted *p*‐value < 0.05. The customized R scripts used for differential expression analysis and data processing are provided in the Supporting Information.

### 2.2 Prediction of circRNA–miRNA–mRNA Interactions and Construction of ceRNA Regulatory Network

To function as a ceRNA, circRNAs sequester specific miRNAs through their corresponding miRNA response elements (MREs). To identify potential circRNA‐miRNA interactions, predicted target miRNAs were obtained from the Cancer‐Specific CircRNA Database (CSCD, version 1.0, accessed on March, 2023; http://gb.whu.edu.cn/CSCD/), which is an online functional database for circRNAs. These predicted targets were termed CTmiRNAs (CSCD target miRNAs). CTmiRNAs underwent additional screening based on the DEmiRNAs derived from the TCGA database. To identify possible target miRNAs for DEcircRNAs, an intersection was conducted between CTmiRNAs and significantly downregulated miRNAs from TCGA. Subsequently, the overlapped miRNAs of these two algorithms were referred to as final shared miRNAs (FS‐miRNAs).

miRTarBase [20] (release 9.0, accessed March 2023; https://awi.cuhk.edu.cn/~miRTarBase/miRTarBase_2025/php/index.php) is a comprehensive repository that compiles experimentally validated miRNA‐target interactions, providing a pivotal resource for advancing research in the field of miRNA biology and regulatory networks. The multiMiR package [21] (V 1.20.0) in R was used for the identification of miRNA target genes based on data from the miRTarBase database. The genes found to be associated with FS‐miRNAs were named final mRNAs (FmRNAs). To identify genes that are potentially involved in BC, we integrated the results from the miRTarBase database, which were referred to as FmRNAs, with the upregulated DEgenes identified through TCGA data analysis. The resultant functional genes were designated as final shared mRNAs (FS‐mRNAs).

Based on the ceRNA hypothesis, a regulatory network was constructed by integrating all preceding steps, including the identification of differentially expressed RNAs and the prediction of circRNA‐miRNA‐mRNA interactions. These sequential analyses collectively provided the input for the circRNA/miRNA/mRNA regulatory network, which was subsequently visualized using Cytoscape software (V 3.10.0) [22].

### 2.3 BC Tissue Sampling, Total RNA Extraction, and RT‐qPCR

The study was approved by the Medical Ethics Committee of Babol University of Medical Sciences (Ethical code: IR.MUBABOL.HRI.REC.1401.248), and all patients signed the written informed consents prior to operation. Cancer tissues and adjacent normal tissues of 24 patients who had undergone surgery between May 2023 and September 2024 were collected. No chemotherapy or radiation was administered before the operation for any of the patients with BC. Tissues were collected using RNA later solution and then stored in a −80°C refrigerator for further use.

To experimentally validate candidate circRNAs, total RNA was extracted from BC and adjacent non‐tumor tissues with TRIzol reagent (RNX‐ Plus; Sinaclon, Iran). cDNA was synthesized with the Reverse Transcription kit (YTA‐cDNA Synthesis Kit, Yektatajhiz, Iran). RT‐qPCR was performed with SYBR Green Master Mix (Ampliqon, Denmark) using the Applied Biosystems StepOnePlus instrument. GAPDH was employed as an internal reference, and the relative expression levels of circRNAs were calculated with the 2^−ΔΔCT^ (Livak) method. The sequences of specific primers used in this study are listed in Table 1.

**TABLE 1:** Primer sequences for reverse‐transcription quantitative polymerase chain reaction (RT‐qPCR).

Gene	Primer	Primer sequence (5′–3′)	Amplicon size
hsa_circ_0000231	Forward	GCTCCACTGAACAGATAAGGGT	155
	Reverse	TCCCACTTCTGTCAGCCATT	
hsa_circ_0011385	Forward	TCAACCAGTATAGTGCCAAGGA	87
	Reverse	TTGCCCCCAAAGTCAAAACC	
GAPDH	Forward	AGCCTCAAGATCATCAGCAAT	101
	Reverse	GTCATGAGTCCTTCCACGATAC	

### 2.4 Construction of the Protein‐Protein Interaction Network and Functional Enrichment Analysis

We employed the GeneMANIA plugin (V 3.5.3) [23] within the Cytoscape software (V 3.10.2) to generate a protein‐protein interaction (PPI) network for target genes of DEcircRNAs validated by RT‐qPCR. The gene exhibiting the most statistically significant *p*‐value was chosen as the central node of the interactions. A weighted Gene Ontology (GO) network was created using GeneMANIA to identify genes associated with the molecular mechanism of BC, integrating multiple biological relationships, including co‐expression, co‐localization, shared pathways, physical interactions, genetic interactions, and protein domain similarities. A total of 20 genes were prioritized based on their high connectivity and functional relevance across these criteria and were selected for the establishment of the PPI network.

Furthermore, GO [24] and Kyoto Encyclopedia of Genes and Genomes (KEGG) [25, 26] enrichment analyses were performed to elucidate the functional roles and pathways associated with the genes within the constructed PPI network using the R package clusterProfiler (V 4.12.0) [27]. GO analyses, encompassing BP, cellular component (CC), and molecular function (MF) terms, and KEGG pathway analysis were conducted with the Benjamini‐Hochberg method, applying a threshold of *p*‐value < 0.05 to identify statistically significant functional annotations and enriched pathways.

### 2.5 Statistical Analysis

Results from a minimum of three independent experiments are expressed as the mean ± standard deviation. R (V 4.2.2) and SPSS 27.0 software were used for data processing and statistical analyses. To compare the differences in the expression levels of the candidate circRNAs between BC tissues and adjacent normal tissues, the Wilcoxon signed‐rank test was used, and *p*‐values < 0.05 were considered statistically significant. The association between the circRNA expression and the clinicopathological characteristics of BC patients was evaluated using Spearman's rank correlation coefficient analysis. Receiver operating characteristic (ROC) curve analysis was conducted to evaluate the diagnostic performance of the candidate circRNAs in differentiating BC tissues from adjacent non‐tumor tissues.

## 3 Results and Discussion

### 3.1 Identification of BC‐related RNAs

**FIGURE 2:**
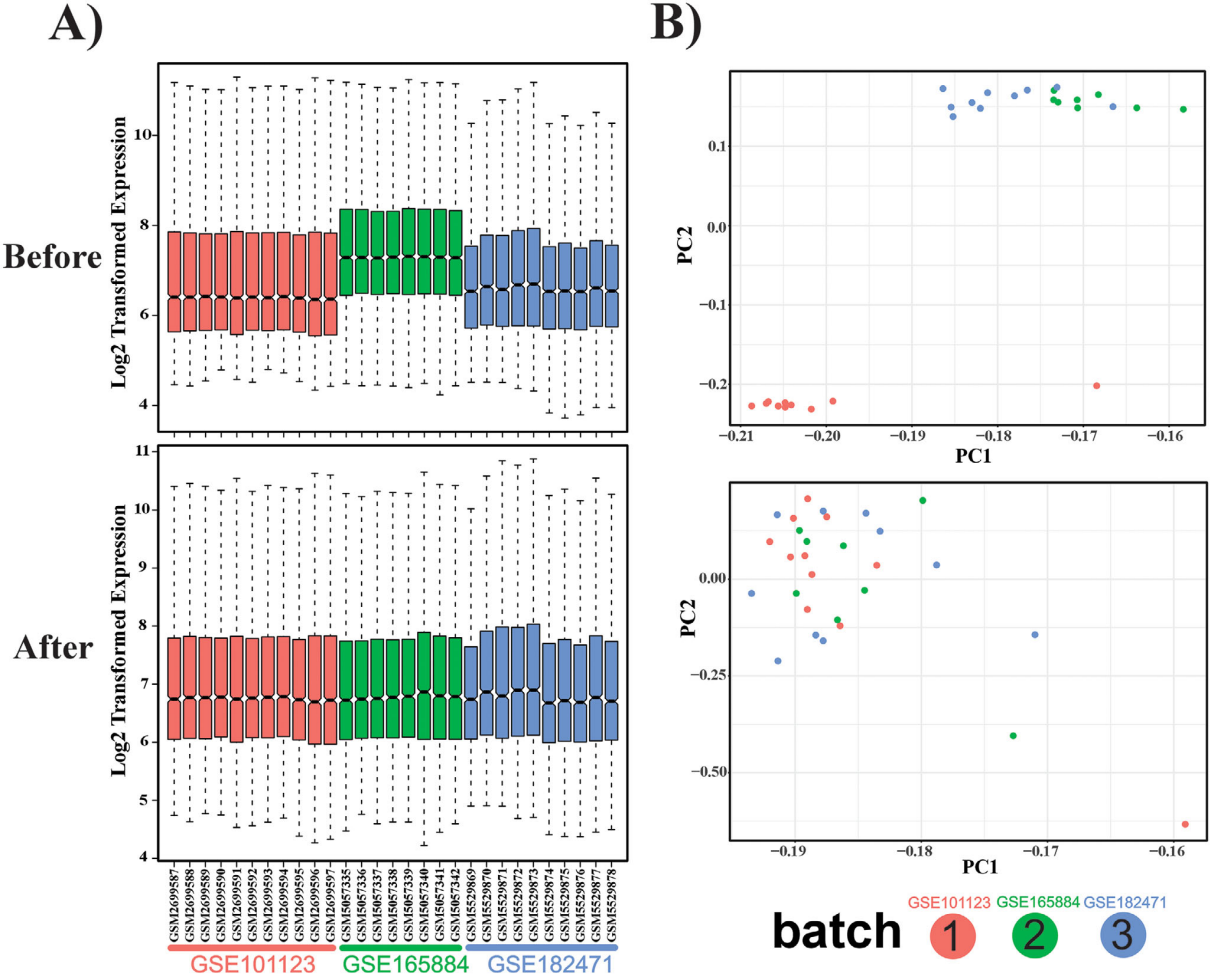
(A) Box plots depict the overall expression profiles of the three gene expression datasets before and after batch effect correction using the ComBat method from the sva package in R. (B) Principal component analysis (PCA) displays the distribution of sample expression values across the three datasets before and after batch effect removal.

**FIGURE 3:**
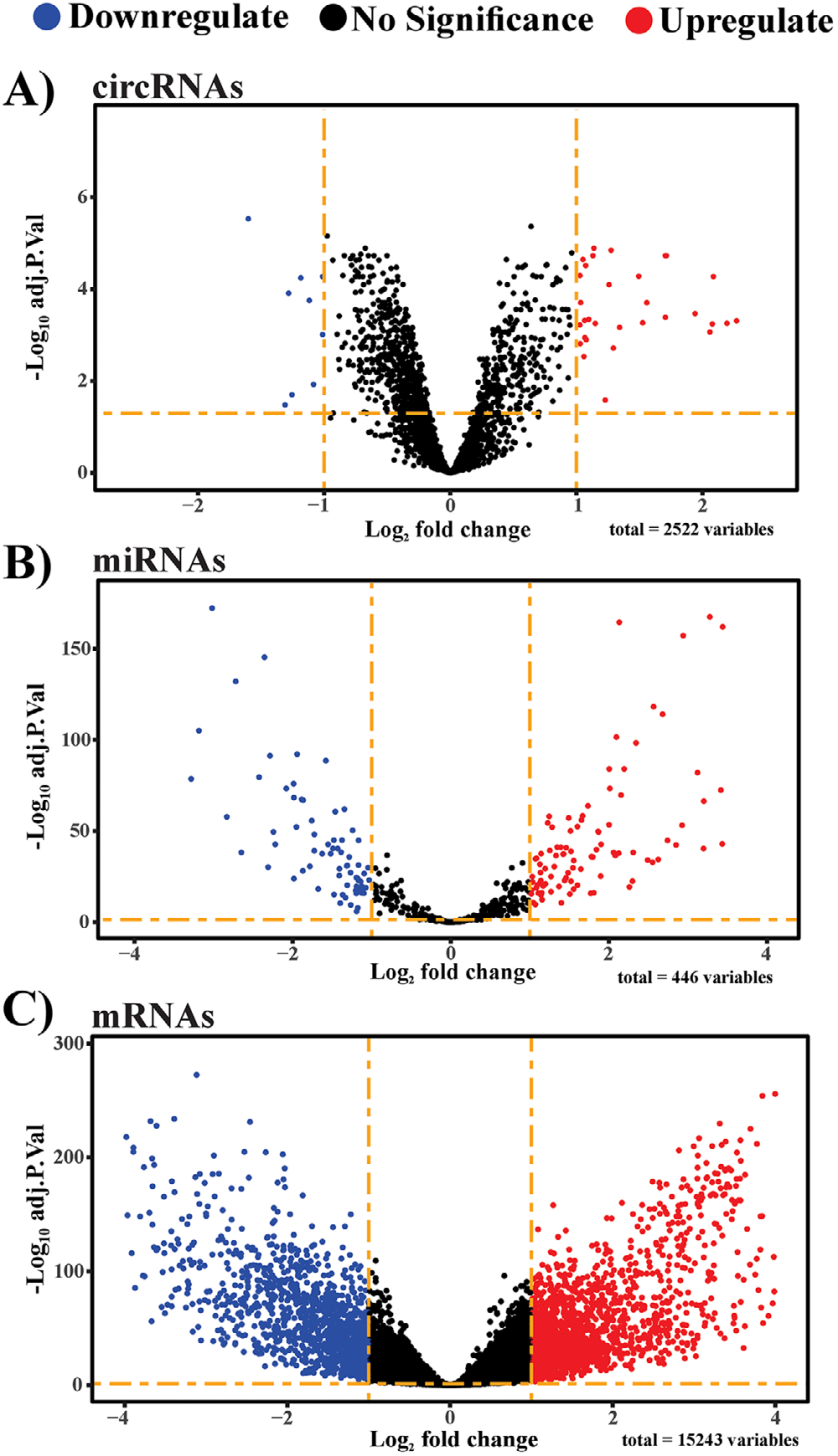
Volcano plots of (A) differentially expressed circular RNAs (DEcircRNAs), (B) differentially expressed miRNAs (DEmiRNAs), and (C) differentially expressed mRNAs (DEgenes). The blue and red dots represent significantly downregulated and upregulated genes, respectively. The black dots indicate RNAs that did not meet the selection criteria of adjusted *p*‐value < 0.05 and |log2Fold Change| > 1.

Initially, examination of the datasets confirmed that all data were already in log scale and required no further normalization. Following the integration of the three datasets, the widely used ComBat function from the R sva package was applied to remove hidden batch effects (Figure 2). To confirm the effectiveness of batch effect correction, PCA plots were generated before and after ComBat adjustment. As shown in Figure 2B, clustering by dataset was minimized after correction. Differential expression analysis was performed using the Limma package, resulting in the identification of 2522 DEcircRNAs. Among these, 40 circRNAs were deemed statistically significant, meeting the criteria of adjusted *p*‐value < 0.05 and |log2FoldChange| > 1. These circRNAs, referred to as DEcircRNAs, were identified in BC samples compared to normal tissues. Of the significant DEcircRNAs, 31 were upregulated, and nine were downregulated (Figure 3A). Following additional analysis and refinement of the 40 identified DEcircRNAs, two upregulated circRNAs, hsa_circ_0000231 and hsa_circ_0011385, were selected as the primary focus for this study. The detailed information regarding the candidate circRNAs is presented in Table 2. These two circRNAs were selected for validation because of their differential expression characteristics (adjusted *p*‐value and log2FoldChange), lack of prior investigation in BC, and potential involvement in ceRNA‐mediated regulatory pathways. The selection was further supported by previous evidence suggesting potential involvement of these circRNAs in cancer‐related processes.

**TABLE 2:** Comprehensive information of candidate circular RNAs (circRNAs).

circRNA	Gene symbol	Adj. *p*‐value	*p*‐value	LogFC	Regulation	Chromosome
hsa_circ_0000231	ARHGAP12	0.0012	0.0001	1.078	up	chr10
hsa_circ_0011385	EIF3I	0.0005	2.89E‐05	1.065	up	chr1

Research to date on hsa_circ_0000231 has demonstrated elevated expression levels of this circRNA in atherosclerosis (AS) [28, 29], colorectal cancer (CRC) [30, 31, 32], cervical cancer [33], and ovarian cancer (OC) [34], while revealing reduced expression in gastric cancer [35]. Evidence from non‐oncological studies has elucidated regulatory functions for hsa_circ_0000231 in processes such as apoptosis, oxidative stress, cellular migration, and inflammation. For instance, investigations in AS have indicated that hsa_circ_0000231 modulates the disease progression through distinct ceRNA networks, suggesting its potential as a novel therapeutic target for addressing endothelial damage in AS pathology and treatment [28, 29].

Concerning hsa_circ_0011385, a number of investigations have explored the role of this circRNA in various malignancies, including hepatocellular carcinoma [36, 37], thyroid cancer [38, 39], cervical cancer [40, 41], and CRC [42]. These studies have consistently reported an elevated expression of hsa_circ_0011385, suggesting its potential oncogenic role across different cancer types. Evidence from these studies indicates that hsa_circ_0011385 can exert regulatory influence over critical cancer hallmarks such as invasion, migration, and drug resistance through interactions with proteins and participation in diverse ceRNA networks via intricate molecular mechanisms.

**FIGURE 4:**
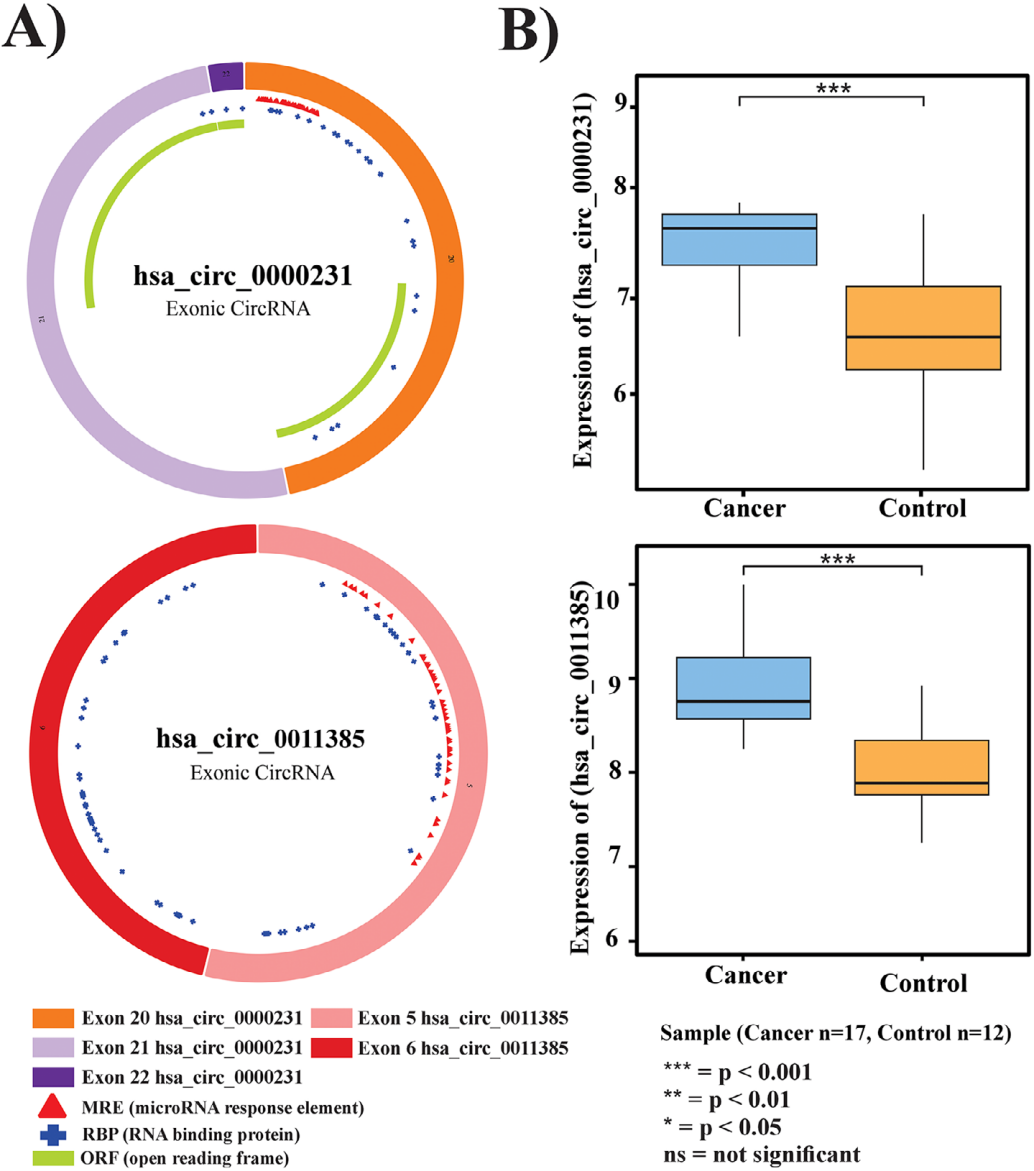
(A) Schematic diagrams illustrate the structure of two candidate differentially expressed circular RNAs (DEcircRNAs), highlighting miRNA response elements (MREs), RNA‐binding protein (RBP) sites, and open reading frames (ORFs). (B) Comparison of the expression levels of candidate DEcircRNAs between tumor and normal control samples based on microarray data. The results of these comparisons indicate that the candidate circRNAs are significantly upregulated in tumor tissues (*p* < 0.001). *** *p* < 0.001; ** *p* < 0.01; * *p* < 0.05; ns, not significant (cancer samples = 17, control samples = 12).

In the subsequent phase of constructing the circRNA/miRNA/mRNA network, we aimed to identify DEmiRNAs and DEgenes. Using the DESeq2 package in R, data from the TCGA‐BRCA database were analyzed to verify DEmiRNAs and DEgenes by comparing primary tumor tissues with normal solid tissues. The results indicated the presence of 48 upregulated and 28 downregulated DEmiRNAs (Figure 3B), alongside 1620 upregulated and 1211 downregulated DEgenes (Figure 3C). Differential expression was verified based on the thresholds of |log2FoldChange| > 1 and adjusted *p*‐value < 0.05. The structure and expression levels of the two candidate DEcircRNAs in tumor and normal samples from microarray datasets are shown in Figure 4.

### 3.2 Construction of circRNA/miRNA/mRNA Regulatory Network in BC

**FIGURE 5:**
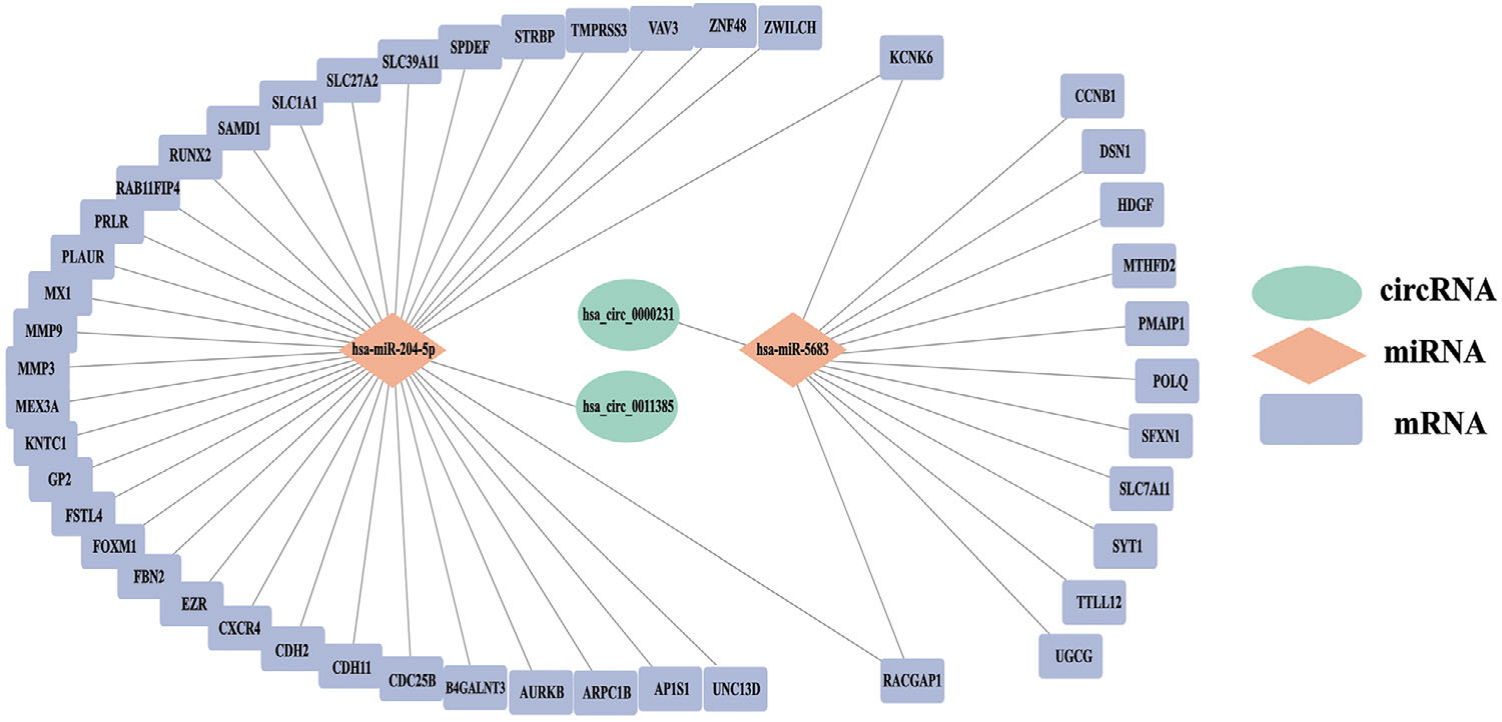
Preliminary circRNA/miRNA/mRNA regulatory network in breast cancer (BC). The competing endogenous RNA (ceRNA) network comprises two circular RNAs (circRNAs) (hsa_circ_0000231, hsa_circ_0011385), two micro RNAs (miRNAs) (hsa‐miR‐5683 and hsa‐miR‐204‐5p), and 46 messenger RNAs (mRNAs).

CircRNAs have been increasingly recognized for their role as ceRNAs in various cancers, functioning as molecular sponges that sequester miRNAs, thereby inhibiting miRNA‐mediated degradation or translational repression of target mRNAs. To elucidate the regulatory role of the selected circRNAs in BC, a circRNA/miRNA/mRNA interaction network was constructed. To minimize random associations, only circRNA–miRNA–mRNA interactions predicted by CSCD and supported by TCGA expression data were retained. This stringent filtering resulted in a focused network with two high‐confidence miRNA nodes, which strengthens its reliability compared to background random pairing. Both candidate DEcircRNAs of this study harbor MRE sites for miRNA sponge (Figure 4A). To determine the target miRNAs of the selected DEcircRNAs, the CSCD database predicted 61 miRNAs for hsa_circ_0000231 and 59 miRNAs for hsa_circ_0011385, which were each independently intersected with 28 downregulated miRNAs from the TCGA‐BRCA dataset to identify overlapping miRNAs. Further investigation into the downstream effects of this sponging activity involved the use of the miRTarBase database to identify mRNAs with complementary binding sites to the FS‐miRNAs. These target mRNAs, referred to as FmRNAs, were intersected with the upregulated DEgenes, collectively yielding a total of 13 genes for hsa_circ_0000231 and 35 genes for hsa_circ_0011385. Based on the established circRNA‐miRNA and miRNA‐mRNA interactions, a regulatory network was constructed, consisting of two circRNA nodes, two miRNA nodes, and 46 mRNA nodes. Detailed bioinformatic results related to network construction are provided in Table 3, and the corresponding ceRNA network is illustrated in Figure 5. Notably, RACGAP1 and KCNK6 were identified as common targets of both miRNAs within this network.

**TABLE 3:** Detailed results of the bioinformatic analyses used for the construction of the competing endogenous RNA (ceRNA) network.

circRNA	CSCD	Merge CSCD‐TCGA	Final shared miRNAs (FS‐miRNAs)	multiMiR (miRTarBase)	Merge miRTarBase‐TCGA	Final shared mRNAs (FS‐mRNAs)
hsa_circ_0000231	61 miRNAs	Merge 61 with 28 downregulated miRNAs	One miRNA (**hsa‐miR‐5683**)	98 mRNAs	Merge 98 with 1620 upregulated mRNAs	13 mRNAs
hsa_circ_0011385	59 miRNAs	Merge 59 with 28 downregulated miRNAs	One miRNA (**hsa‐miR‐204‐5p**)	418 mRNAs	Merge 418 with 1620 upregulated mRNAs	35 mRNAs

The ceRNA network provides a mechanistic framework that moves the analysis beyond expression profiling alone. The identification of shared downstream targets also implies that distinct circRNAs may converge on overlapping biological pathways, supporting the idea of coordinated regulatory control in tumor biology. In this context, the network offers not only candidate interactions but also a biologically plausible route for functional interpretation.

Numerous studies across various cancer types have investigated the role of hsa_circ_0000231 in tumorigenesis and cancer progression by evaluating its expression in tissues and cell lines, as well as its interactions with specific miRNAs and proteins [30, 31, 32, 33, 34, 35]. These investigations have suggested that hsa_circ_0000231 may modulate cellular processes such as proliferation and apoptosis through interactions with a range of molecular targets, particularly diverse miRNAs. For instance, Zhang et al. found that hsa_circ_0000231 may exert a dual regulatory role in CRC progression. Firstly, it may function as a ceRNA by sponging miR‐375, thereby upregulating CCND2 expression. Secondly, hsa_circ_0000231 may enhance CRC progression by interacting with the IGF2BP3 protein, stabilizing CCND2, and preventing its degradation [32]. Liu et al. showed that in OC, hsa_circ_0000231 modulates RAP1B expression by acting as a molecular sponge for miR‐140. This regulatory mechanism contributes to enhanced paclitaxel resistance and promotes OC progression [34]. In contrast to findings reported in other malignancies, Fang et al. observed a downregulation of hsa_circ_0000231 in gastric cancer tissues, serum samples, and cell lines. Notably, the expression levels of this circRNA were found to increase in serum following surgical intervention [35]. This divergence underscores the complex and context‐dependent nature of circRNA biology, suggesting that the functional roles of specific circRNAs may vary substantially across different types of cancer and physiological conditions.

### 3.3 Validation of Candidate circRNAs Using RT‐qPCR

**FIGURE 6:**
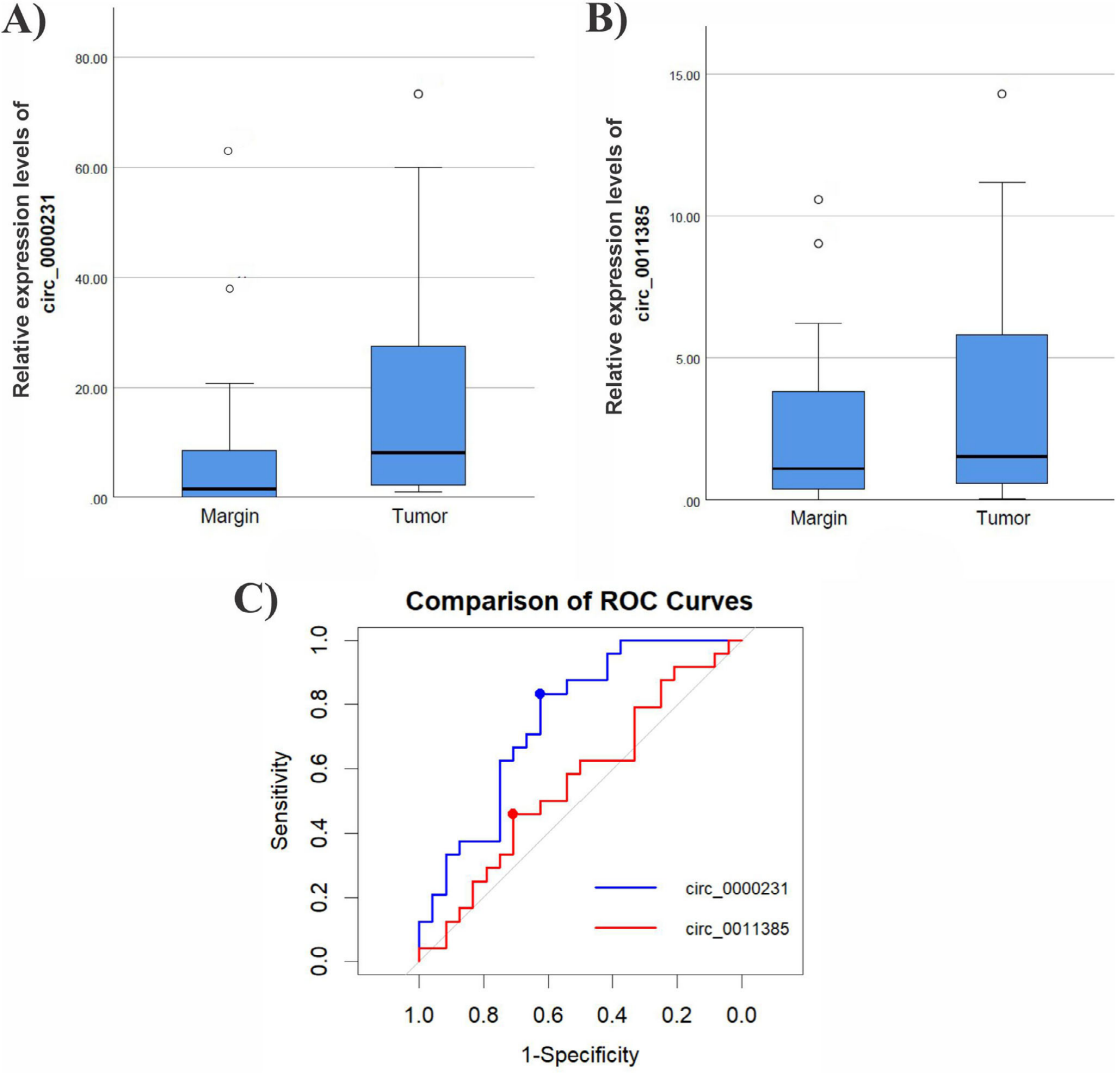
Primary validation of candidate circular RNAs (circRNAs) in breast cancer (BC) tissues by reverse‐transcription quantitative polymerase chain reaction (RT‐qPCR). (A) The expression levels of hsa_circ_0000231 were significantly upregulated in BC tissues compared to adjacent non‐tumor tissues (*p* = 0.005, 95% confidence interval [CI]: 1.365–25.380). (B) The relative expression of hsa_circ_0011385 showed no statistically significant change between BC tissues and adjacent normal tissues (*p* = 0.637, 95% CI: ‐2.295–3.210). (C) Receiver operating characteristic (ROC) curve analysis of hsa_circ_0000231 (AUC = 0.752, *P* < 0.001) and hsa_circ_0011385 (AUC = 0.559, *p* = 0.410). AUC: area under the curve.

To confirm the transcriptional profile analysis results for the two candidate circRNAs, their expression levels were evaluated in 24 pairs of BC tissues and corresponding adjacent normal tissues using RT‐qPCR. The expression levels of hsa_circ_0000231 were significantly upregulated in BC tissues (*p* = 0.005, 95% confidence interval [CI]: 1.365–25.380; Figure 6A). However, hsa_circ_0011385 exhibited no significant difference in expression levels between tumor tissues and adjacent non‐cancerous tissues (*p* = 0.637, 95% CI: ‐2.295–3.210; Figure 6B). Additionally, we performed ROC curve analysis to evaluate the potential of candidate circRNAs as biomarkers for BC diagnosis. ROC curve revealed that hsa_circ_0000231 displayed moderate discriminative ability in distinguishing BC tissues from paired adjacent normal tissues, with an area under the curve (AUC) of 0.752 (*p* < 0.001, 95% CI: 0.612–0.891). In contrast, hsa_circ_0011385 showed poor performance in differentiating BC tissues from adjacent normal tissues, with an AUC of 0.559 (*p* = 0.410, 95% CI: 0.393–0.725; Figure 6C). Furthermore, Spearman's rank correlation coefficient analysis demonstrated a significant positive correlation between the tumor hsa_circ_0000231 expression and tumor grade (*p* = 0.005), T stage (*p* = 0.034), and N stage (*p* = 0.009) in BC patients (Table 4). Consequently, our findings indicate that hsa_circ_0000231 may play a potential oncogenic role in BC, which, according to bioinformatic analyses, can be exerted through the predicted ceRNA network. Further investigations employing a larger sample size, comprehensive clinical follow‐up data, and advanced molecular techniques, such as dual‐luciferase reporter assays and RNA immunoprecipitation, alongside in vitro and in vivo experiments, could provide greater clarity regarding the diagnostic and prognostic potential and the role of this circRNA in BC tumorigenesis and progression. In addition, based on the results of ROC curve analysis, hsa_circ_0000231 demonstrated a relatively good AUC value for differentiating BC tumors from corresponding non‐cancerous tissues. Although the widespread upregulation of hsa_circ_0000231 across various cancers may reduce its tumor‐type specificity, this feature does not lessen its potential clinical value. Rather, it suggests that hsa_circ_0000231 may serve as a broad‐spectrum cancer‐associated marker linked to shared oncogenic pathways. Importantly, its diagnostic specificity could be enhanced through incorporation into multi‐marker panels or integration with established clinical indicators.

**TABLE 4:** Correlation between tumor hsa_circ_0000231 expression levels and the clinicopathological characteristics of patients with breast cancer.

Characteristics	Classification	Number (%)	*p*‐value	*r* (Correlation coefficient)
Age	60≥	11 (45.8%)	0.381	−0.187
	>60	13 (54.2%)		
BMI	18.5–24.9 (normal)	3 (12.5%)	0.127	−0.321
	25–29.9 (overweight)	13 (54.2%)		
	30≤ (obese)	8 (33.3%)		
Tumor size	2 cm≥	4 (16.7%)	0.254	0.242
	2.1–5 cm	17 (70.8%)		
	>5 cm	3 (12.5%)		
Grade	G1	4 (16.7%)	0.005*	0.558*
	G2	13 (54.2%)		
	G3	7 (29.2%)		
T stage	T1	4 (16.7%)	0.034*	0.433*
	T2	14 (58.3%)		
	T3	2 (8.3%)		
	T4	3 (12.5%)		
N stage	N0	15 (62.5%)	0.009*	0.521*
	N1	2 (8.3%)		
	N2	5 (20.8%)		
	N3	2 (8.3%)		
TNM stage	I	3 (12.5%)	0.051	0.402
	II	13 (54.2%)		
	III	7 (29.2%)		

^*^Statistically significant.

In alignment with the methodology of the present study, two independent investigations employed bioinformatics tools to analyze the expression profiles of circRNAs in pancreatic ductal adenocarcinoma (PDAC) and bladder cancer. Following the identification of significantly DEcircRNAs in microarray datasets, these studies validated their expression levels via RT‐qPCR in tumor specimens relative to adjacent normal tissues. Wu et al. reported that hsa_circ_0011385 was upregulated in PDAC and also exhibited a positive correlation with TNM stage and an inverse correlation with overall survival [43]. Lu et al. found a downregulation of hsa_circ_0011385 in bladder cancer based on preliminary microarray analysis. However, subsequent RT‐qPCR validation revealed a considerable overexpression of this circRNA in tumor tissues, highlighting a potential discrepancy between bioinformatics predications and experimental validation [44]. To date, the study by Hu et al. was the sole investigation into the role of hsa_circ_0011385 in BC. This research examined the expression and function of hsa_circ_0011385 in the MCF‐7 BC cell line, revealing elevated levels of this circRNA in cisplatin‐resistant MCF‐7 cells (MCF‐7/DDP). The findings suggested that hsa_circ_0011385 may enhance cisplatin resistance in BC cells by targeting miR‐615‐5p, implicating its potential role in chemoresistance pathways [45]. All previously mentioned studies, especially the investigation conducted on the BC cell line, consistently reported upregulated expression of hsa_circ_0011385. In contrast, the present study, which is the first to evaluate hsa_circ_0011385 expression in BC tissue samples, did not reveal any statistically significant alteration in its expression levels, despite its notable upregulation in microarray data. This discrepancy may be attributable to biological differences between in vitro cell line models and patient‐derived tissues, as well as tumor heterogeneity and the relatively small sample size of our validation cohort. Further investigation in larger, independent patient populations will be required to clarify the role of hsa_circ_0011385 in BC.

Overall, the experimental validation further supports the potential relevance of hsa_circ_0000231 as a candidate biomarker in BC. Its consistent upregulation, moderate discriminative ability, and association with clinicopathological features together indicate that it may have potential as a BC‐associated biomarker.

### 3.4 PPI and Functional Enrichment Analysis

**FIGURE 7:**
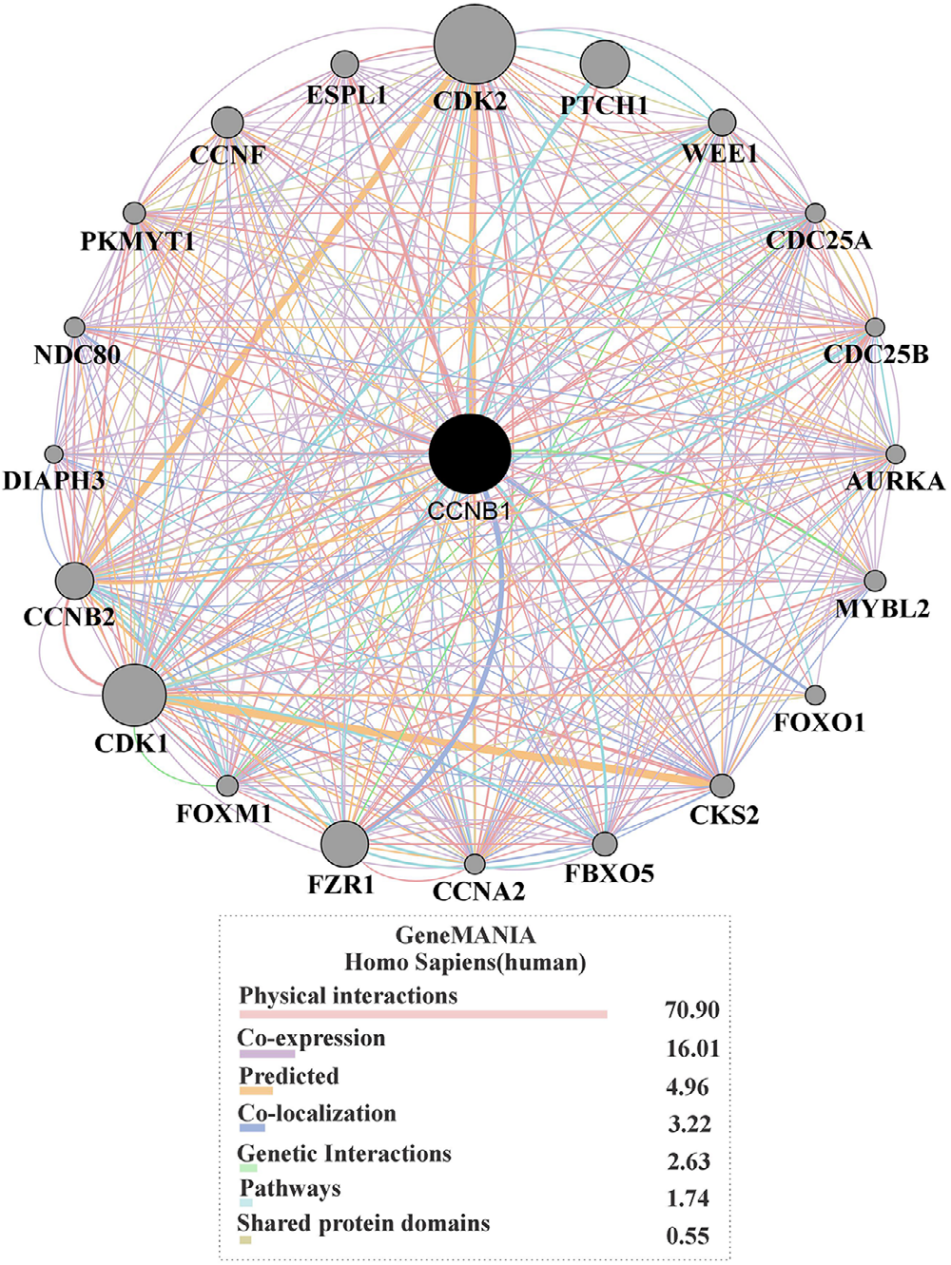
Protein‐protein interaction (PPI) network of Cyclin B1 (CCNB1). The PPI network consists of 21 nodes and 388 edges. Node size reflects the degree value, and edge thickness indicates the strength of supporting data. Different edge colors represent distinct interaction types. The proportions of interaction categories contributing to the network are listed in full below the network diagram.

Cyclin B1 (CCNB1) was selected as the central node for the construction of the PPI network, as it demonstrated the most significant *p*‐value within the set of hsa_circ_0000231‐associated FS‐mRNAs. By utilizing the GeneMANIA plugin in Cytoscape, we generated a PPI network of CCNB1, with edges color‐coded by interaction type and weighted by data source (Figure 7). The resulting network revealed that the nodes were linked via physical interactions (70.90%), co‐expression (16.01%), predicted interactions (4.96%), co‐localization (3.22%), genetic interactions (2.63%), pathways (1.74%), and shared protein domains (0.55%).

**FIGURE 8:**
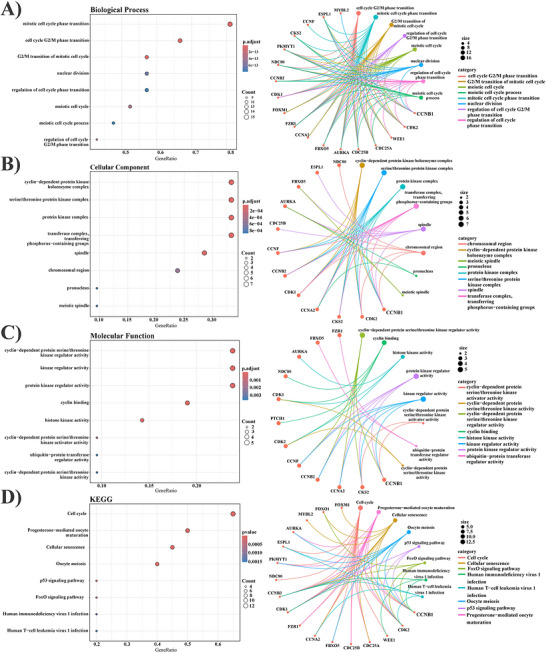
Dot plots and cnetplots of Gene Ontology (GO) and Kyoto Encyclopedia of Genes and Genomes (KEGG) enrichment analyses. The dot plots display significantly enriched terms for (A) biological process (BP), (B) cellular component (CC), (C) molecular function (MF), and (D) KEGG pathways, with terms at the top indicating higher statistical significance. The cnetplots illustrate the association between proteins from the Cyclin B1 (CCNB1) protein‐protein interaction (PPI) network and enriched terms for (A) BP, (B) CC, (C) MF, and (D) KEGG.

To characterize the functional roles of genes within the CCNB1 PPI network, we conducted enrichment analyses using GO terms including BP, CC, and MF categories. In BP analysis, mitotic cell cycle phase transition, cell cycle G2/M phase transition, and nuclear division were the most enriched terms (Figure 8A). In CC terms, the genes were mainly enriched in cyclin‐dependent protein kinase holoenzyme complex, serine/threonine protein kinase complex, protein kinase complex, transferase complex, transferring phosphorus‐containing groups, and spindle (Figure 8B). In MF analysis, the most prominent terms were cyclin‐dependent protein serine/threonine kinase regulator activity, protein kinase regulator activity, kinase regulator activity, cyclin binding, and histone kinase activity (Figure 8C). Furthermore, KEGG pathway enrichment analysis demonstrated that genes interacting with CCNB1 in the PPI network were enriched in key pathways such as cell cycle, progesterone‐mediated oocyte maturation, cellular senescence, and oocyte meiosis (Figure 8D). Enrichment results from GO and KEGG analyses were graphically represented using cnetplots to illustrate the relationships between genes and their associated functional categories and pathways.

These findings support a mechanistic model in which hsa_circ_0000231 may act as a sponge for hsa‐miR‐5683, thereby relieving the repression of CCNB1 and enhancing cell cycle progression. It has been shown that hsa‐miR‐5683 downregulation promotes gastric cancer cell proliferation and inhibits apoptosis, and gene pyruvate dehydrogenase kinase 4 was found as a possible downstream target for this miRNA [46]. Another study reported decreased levels of hsa‐miR‐5683 in all BC subtypes, suggesting it as a favorable prognostic biomarker in BC [47]. Our bioinformatic analyses identified CCNB1 as a potential target of hsa‐miR‐5683. CCNB1, a key member of the cyclin family, is critically involved in cell cycle regulation through interactions with other regulatory molecules. It forms a complex with cyclin‐dependent kinase 1, which is essential for initiation and progression of mitosis, particularly during G2/M transition [48]. A recent meta‐analysis indicated that elevated CCNB1 expression correlates with poorer prognosis in BC patients. This association may be attributed to its role in promoting cell cycle progression and mitotic activity in tumor cells [49]. In addition, suppression of CCNB1 has been shown to enhance the chemosensitivity of BC cells, suggesting its potential as a therapeutic target [50]. The enrichment of multiple cell cycle‐ related pathways observed in this study further reinforces the idea that hsa_circ_0000231 may contribute to tumor progression through dysregulation of mitotic control. Consequently, the proposed hsa_circ_0000231/ hsa‐miR‐5683/ CCNB1 axis provides a plausible mechanistic explanation for the oncogenic potential of this candidate circRNA. Collectively, these findings support the potential involvement of the hsa_circ_0000231‐associated regulatory network in BC progression and provide a basis for future functional investigations.

## 4 Conclusion

This study applied an integrated bioinformatic and experimental strategy to identify circRNAs with potential diagnostic and prognostic relevance in BC. Among the candidates, one circRNA (hsa_circ_0000231) demonstrated consistent overexpression and moderate discriminative ability between tumor and normal tissues, supporting its potential as a biomarker. Importantly, this research highlights a structured analytical framework that combines multi‐dataset screening, candidate prioritization, functional analysis, and experimental validation, thereby improving the reliability of biomarker identification. However, future studies involving larger cohorts and functional investigations are needed to validate these findings and clarify the biological role of the identified circRNA. In addition, the predicted hsa_circ_0000231/hsa‐miR‐5683/CCNB1 regulatory axis deserves further investigation, which could provide deeper insight into the molecular mechanisms underlying BC tumorigenesis and progression.

## Author Contributions

**MohamadReza Hashemi**: investigation, methodology, validation, and writing – original draft. **Pejman Morovat**: data curation, software, and visualization. **Azin Khoshghiafeh**: resources and writing – review and editing. **Novin Nikbakhsh**: resources. **Mohamadreza Ahmadifard**: supervision, project administration, and funding acquisition. All authors reviewed and approved the final version of the manuscript.

## Funding

This research was funded by the Deputy of Research and Technology of Babol University of Medical Sciences, grant number 724134480.

## Ethics Statement

This study was approved by the Medical Ethics Committee of Babol University of Medical Science (Ethical code: IR.MUBABOL.HRI.REC.1401.248). All procedures involving human participants were conducted in accordance with the Declaration of Helsinki.

## Conflicts of Interest

The authors declare no conflicts of interest.

## Supporting information




**Supporting File**: ansa70098‐sup‐0001‐SuppMat.pdf.

## Data Availability

The datasets analyzed in this study are publicly available in the NCBI Gene Expression Omnibus (GEO) database (http://www.ncbi.nlm.nih.gov/geo/) under GSE101123, GSE165884, and GSE182471 accession numbers and in the Cancer Genome Atlas Breast Invasive Carcinoma project (TCGA‐BRCA, https://portal.gdc.cancer.gov/projects/TCGA‐BRCA). Customized R scripts used for data analysis are provided in the Supporting Information. The remainder of the data that support the findings of this study are available from the corresponding author upon reasonable request. These data are not publicly available due to privacy and ethical restrictions.

## References

[1] F. Bray , M. Laversanne , H. Sung , et al., “Global Cancer Statistics 2022: GLOBOCAN Estimates of Incidence and Mortality Worldwide for 36 Cancers in 185 Countries,” CA: A Cancer Journal for Clinicians 74, no. 3 (2024): 229–263, 10.3322/caac.21834. 38572751

[2] N. Pashayan , A. C. Antoniou , U. Ivanus , et al., “Personalized Early Detection and Prevention of Breast Cancer: ENVISION Consensus Statement,” Nature Reviews Clinical Oncology 17, no. 11 (2020): 687–705, 10.1038/s41571-020-0388-9. PMC7567644 32555420

[3] Y. Liang , H. Zhang , X. Song , and Q. Yang , “Metastatic Heterogeneity of Breast Cancer: Molecular Mechanism and Potential Therapeutic Targets,” Seminars in Cancer Biology 60 (2020): 14–27, 10.1016/j.semcancer.2019.08.012. 31421262

[4] E. S. McDonald , A. S. Clark , J. Tchou , P. Zhang , and G. M. Freedman , “Clinical Diagnosis and Management of Breast Cancer,” Journal of Nuclear Medicine 57, no. Suppl 1 (2016): 9S–16S, 10.2967/jnumed.115.157834. 26834110

[5] L. S. Kristensen , M. S. Andersen , L. V. W. Stagsted , K. K. Ebbesen , T. B. Hansen , and J. Kjems , “The Biogenesis, Biology and Characterization of Circular RNAs,” Nature Reviews Genetics 20, no. 11 (2019): 675–691, 10.1038/s41576-019-0158-7. 31395983

[6] M. Shahpari , M. Hashemi , T. Younesirad , A. Hasanzadeh , M. M. Mosanne , and M. Ahmadifard , “The Functional Roles of Competitive Endogenous RNA (ceRNA) Networks in Apoptosis in Human Cancers: The circRNA/miRNA/mRNA Regulatory Axis and Cell Signaling Pathways,” Heliyon 10, no. 21 (2024): e37089, 10.1016/j.heliyon.2024.e37089. 39524849 PMC11546195

[7] V. M. Conn , A. M. Chinnaiyan , and S. J. Conn , “Circular RNA in Cancer,” Nature Reviews Cancer 24, no. 9 (2024): 597–613, 10.1038/s41568-024-00721-7. 39075222

[8] L. Salmena , L. Poliseno , Y. Tay , L. Kats , and P. P. Pandolfi , “A ceRNA Hypothesis: The Rosetta Stone of a Hidden RNA Language?” Cell 146, no. 3 (2011): 353–358, 10.1016/j.cell.2011.07.014. 21802130 PMC3235919

[9] L. Chen and G. Shan , “CircRNA in Cancer: Fundamental Mechanism and Clinical Potential,” Cancer Letters 505 (2021): 49–57, 10.1016/j.canlet.2021.02.004. 33609610

[10] X. Huang , C. Song , J. Zhang , L. Zhu , and H. Tang , “Circular RNAs in Breast Cancer Diagnosis, Treatment and Prognosis,” Oncology Research 32, no. 2 (2023): 241–249, 10.32604/or.2023.046582. 38186573 PMC10765117

[11] Y. Yao , X. Li , L. Cheng , X. Wu , and B. Wu , “Circular RNA FAT atypical Cadherin 1 (circFAT1)/microRNA‐525‐5p/Spindle and Kinetochore‐associated Complex Subunit 1 (SKA1) Axis Regulates Oxaliplatin Resistance in Breast Cancer by Activating the Notch and Wnt Signaling Pathway,” Bioengineered 12, no. 1 (2021): 4032–4043, 10.1080/21655979.2021.1951929. 34288822 PMC8806415

[12] L. Ding and Z. Xie , “CircWHSC1 regulates Malignancy and Glycolysis by the miR‐212‐5p/AKT3 Pathway in Triple‐negative Breast Cancer,” Experimental and Molecular Pathology 123 (2021): 104704, 10.1016/j.yexmp.2021.104704. 34624276

[13] T. Barrett , S. E. Wilhite , P. Ledoux , et al., “NCBI GEO: Archive for Functional Genomics Data Sets–update,” Nucleic Acids Research 41, no. Database issue (2013): D991–5, 10.1093/nar/gks1193. PMC3531084 23193258

[14] M. Deng , J. Brägelmann , J. L. Schultze , and S. Perner , “Web‐TCGA: An Online Platform for Integrated Analysis of Molecular Cancer Data Sets,” BMC Bioinformatics [Electronic Resource] 17, no. 1 (2016): 72, 10.1186/s12859-016-0917-9. 26852330 PMC4744375

[15] J. T. Leek , W. E. Johnson , H. S. Parker , A. E. Jaffe , and J. D. Storey , “The sva Package for Removing Batch Effects and Other Unwanted Variation in High‐throughput Experiments,” Bioinformatics 28, no. 6 (2012): 882–883, 10.1093/bioinformatics/bts034. 22257669 PMC3307112

[16] M. E. Ritchie , B. Phipson , D. Wu , et al., “Limma Powers Differential Expression Analyses for RNA‐sequencing and Microarray Studies,” Nucleic Acids Research 43, no. 7 (2015): e47, 10.1093/nar/gkv007. 25605792 PMC4402510

[17] R. Li , H. Qu , S. Wang , et al., “GDCRNATools: An R/Bioconductor Package for Integrative Analysis of lncRNA, miRNA and mRNA Data in GDC,” Bioinformatics 34, no. 14 (2018): 2515–2517, 10.1093/bioinformatics/bty124. 29509844

[18] M. D. Robinson and A. Oshlack , “A Scaling Normalization Method for Differential Expression Analysis of RNA‐seq Data,” Genome Biology 11, no. 3 (2010): R25, 10.1186/gb-2010-11-3-r25. 20196867 PMC2864565

[19] C. W. Law , Y. Chen , W. Shi , and G. K. Smyth , “voom: Precision Weights Unlock Linear Model Analysis Tools for RNA‐seq Read Counts,” Genome Biology 15, no. 2 (2014): R29, 10.1186/gb-2014-15-2-r29. 24485249 PMC4053721

[20] S. Cui , S. Yu , H.‐Y. Huang , et al., “miRTarBase 2025: Updates to the Collection of Experimentally Validated microRNA–target Interactions,” Nucleic Acids Research 53, no. D1 (2025): D147–D156, 10.1093/nar/gkae1072. 39578692 PMC11701613

[21] Y. Ru , K. J. Kechris , B. Tabakoff , et al., “The multiMiR R Package and Database: Integration of microRNA–target Interactions Along With Their Disease and Drug Associations,” Nucleic Acids Research 42, no. 17 (2014): e133–e133, 10.1093/nar/gku631. 25063298 PMC4176155

[22] P. Shannon , A. Markiel , O. Ozier , et al., “Cytoscape: A Software Environment for Integrated Models of Biomolecular Interaction Networks,” Genome Research 13, no. 11 (Nov 2003): 2498–2504, 10.1101/gr.1239303. 14597658 PMC403769

[23] M. Franz , H. Rodriguez , C. Lopes , et al., “GeneMANIA Update 2018,” Nucleic Acids Research 46, no. W1 (2018): W60–W64, 10.1093/nar/gky311. 29912392 PMC6030815

[24] T. G. O. Consortium , S. A. Aleksander , J. Balhoff , et al., “The Gene Ontology Knowledgebase in 2023,” Genetics 224, no. 1 (2023): iyad031, 10.1093/genetics/iyad031. 36866529 PMC10158837

[25] M. Kanehisa , M. Furumichi , Y. Sato , Y. Matsuura , and M. Ishiguro‐Watanabe , “KEGG: Biological Systems Database as a Model of the Real World,” Nucleic Acids Research 53, no. D1 (2025): D672–D677, 10.1093/nar/gkae909. 39417505 PMC11701520

[26] M. Kanehisa , “KEGG: Kyoto encyclopedia of Genes and Genomes,” Nucleic Acids Research 28, no. 1 (2000): 27–30, 10.1093/nar/28.1.27. 10592173 PMC102409

[27] T. Wu , E. Hu , S. Xu , et al., “clusterProfiler 4.0: A Universal Enrichment Tool for Interpreting Omics Data,” Innovation 2, no. 3 (2021): 100141, 10.1016/j.xinn.2021.100141. 34557778 PMC8454663

[28] X. Shao , Z. Liu , S. Liu , N. Lin , and Y. Deng , “Astragaloside IV Alleviates Atherosclerosis Through Targeting circ_0000231/miR‐135a‐5p/CLIC4 Axis in AS Cell Model in Vitro,” Molecular and Cellular Biochemistry 476, no. 4 (2021): 1783–1795, 10.1007/s11010-020-04035-8. 33439448

[29] Z. Chen , J. Zhao , S. Wang , and Q. Li , “Tanshinone IIA Attenuates Ox‐LDL ‐Induced Endothelial Cell Injury by Inhibiting NF‐kappa B Pathway via circ_0000231/miR‐590‐5p/TXNIP Axis,” Chemical Biology & Drug Design 103, no. 1 (2024): e14394, 10.1111/cbdd.14394. 37955049

[30] Y. Liu , H. Li , X. Ye , et al., “Hsa_circ_0000231 knockdown Inhibits the Glycolysis and Progression of Colorectal Cancer Cells by Regulating miR‐502‐5p/MYO6 Axis,” World Journal of Surgical Oncology 18, no. 1 (2020): 255, 10.1186/s12957-020-02033-0. 32993655 PMC7526375

[31] J. Wang , S. Li , G. Zhang , and H. Han , “Sevoflurane Inhibits Malignant Progression of Colorectal Cancer via hsa_circ_0000231‐mediated miR‐622,” Journal of Biological Research 28, no. 1 (2021): 14, 10.1186/s40709-021-00145-6. 34183076 PMC8237491

[32] W. Zhang , B. Wang , Y. Lin , et al., “hsa_circ_0000231 Promotes Colorectal Cancer Cell Growth Through Upregulation of CCND2 by IGF2BP3/miR‐375 Dual Pathway,” Cancer Cell International 22, no. 1 (2022): 27, 10.1186/s12935-022-02455-8. 35033075 PMC8760675

[33] F. Ji , Y. Lu , S. Chen , et al., “IGF2BP2‐modified Circular RNA circARHGAP12 Promotes Cervical Cancer Progression by Interacting m6A/FOXM1 Manner,” Cell Death Discovery 7, no. 1 (2021): 215, 10.1038/s41420-021-00595-w. 34392306 PMC8364552

[34] J. Liu , H. Wang , S. Xiao , S. Zhang , Y. Qi , and M. Wang , “Circ_0000231 promotes Paclitaxel Resistance in Ovarian Cancer by Regulating miR‐140/RAP1B,” American Journal of Cancer Research 13, no. 3 (2023): 872–885. 37034216 PMC10077041

[35] R. Fang , W. Yuan , C. Mao , et al., “Human Circular RNA hsa_circ_0000231 Clinical Diagnostic Effectiveness as a New Tumor Marker in Gastric Cancer,” Cancer Reports 7, no. 5 (2024): e2081, 10.1002/cnr2.2081. 38703060 PMC11069127

[36] D. Fu , Q. Ji , C. Wang , L. Yu , and R. Yu , “Aloin Decelerates the Progression of Hepatocellular Carcinoma Through circ_0011385/miR‐149‐5p/WT1 Axis,” Cell Cycle 20, no. 23 (2021): 2476–2493, 10.1080/15384101.2021.1988227. 34720052 PMC8794511

[37] C. Ni , S. Yang , Y. Ji , et al., “Hsa_circ_0011385 knockdown Represses Cell Proliferation in Hepatocellular Carcinoma,” Cell Death Discovery 7, no. 1 (2021): 270, 10.1038/s41420-021-00664-0. 34599150 PMC8486831

[38] F. Xia , Y. Chen , B. Jiang , N. Bai , and X. Li , “Hsa_circ_0011385 accelerates the Progression of Thyroid Cancer by Targeting miR‐361‐3p,” Cancer Cell International 20 (2020): 49, 10.1186/s12935-020-1120-7. 32082079 PMC7017482

[39] X. Yao , H. Liu , Z. Wang , et al., “Circular RNA EIF3I Promotes Papillary Thyroid Cancer Progression by Interacting With AUF1 to Increase Cyclin D1 Production,” Oncogene 42, no. 43 (2023): 3206–3218, 10.1038/s41388-023-02830-3. 37697064

[40] L. Hu , M. Zhou , L. Xue , and J. Zhang , “Circular RNA hsa_circ_0011385 Contributes to Cervical Cancer Progression Through Sequestering miR‐149–5p and Increasing PRDX6 Expression,” Reproductive Biology 22, no. 2 (2022): 100619, 10.1016/j.repbio.2022.100619. 35240453

[41] A. L. Xu , W. S. Wang , M. Y. Zhao , J. N. Sun , X. R. Chen , and J. Q. Hou , “Circular RNA circ_0011385 Promotes Cervical Cancer Progression Through Competitively Binding to miR‐149‐5p and Up‐Regulating SOX4 Expression,” Kaohsiung Journal of Medical Sciences 37, no. 12 (2021): 1058–1068, 10.1002/kjm2.12432. 34369654 PMC11896328

[42] J. Wang , S. Ke , Y. Gong , et al., “Circ_0011385 knockdown Inhibits Cell Proliferation, Migration and Invasion, Whereas Promotes Cell Apoptosis by Regulating miR‐330‐3p/MYO6 Axis in Colorectal Cancer,” Biomedical Journal 46, no. 1 (2023): 110–121, 10.1016/j.bj.2022.01.007. 35091088 PMC10104957

[43] H. Wu , B. Wang , L. Wang , and Y. Xue , “Circular RNAs 0000515 and 0011385 as Potential Biomarkers for Disease Monitoring and Determining Prognosis in Pancreatic Ductal Adenocarcinoma,” Oncology Letters 23, no. 2 (2022): 56, 10.3892/ol.2021.13174. 34992688 PMC8721853

[44] H.‐C. Lu , J.‐Q. Yao , X. Yang , et al., “Identification of a Potentially Functional circRNA–miRNA–mRNA Regulatory Network for Investigating Pathogenesis and Providing Possible Biomarkers of Bladder Cancer,” Cancer Cell International 20 (2020): 31, 10.1186/s12935-020-1108-3. 32015691 PMC6990554

[45] Y. Hu , X. Ye , F. Xiang , L. Chen , and P. Chen , “Circ_0011385 increases Cisplatin Resistance in Breast Cancer via miR‐615‐5p Suppression,” Tropical Journal of Pharmaceutical Research 23, no. 2 (2024): 243–249, 10.4314/tjpr.v23i2.2.

[46] Y. Miao , Q. Li , G. Sun , et al., “MiR‐5683 Suppresses Glycolysis and Proliferation Through Targeting Pyruvate Dehydrogenase Kinase 4 in Gastric Cancer,” Cancer Medicine 9, no. 19 (2020): 7231–7243, 10.1002/cam4.3344. 32780563 PMC7541129

[47] B. Y. Abohalawa , H. Shaath , R. Elango , et al., “MicroRNAome Profiling of Breast Cancer Unveils Hsa‐miR‐5683 as a Tumor Suppressor microRNA Predicting Favorable Clinical Outcome,” Cancer Cell International 24, no. 1 (2024): 377, 10.1186/s12935-024-03550-8. 39538254 PMC11562357

[48] Y. Fang , H. Yu , X. Liang , J. Xu , and X. Cai , “Chk1‐induced CCNB1 Overexpression Promotes Cell Proliferation and Tumor Growth in Human Colorectal Cancer,” Cancer Biology & Therapy 15, no. 9 (2014): 1268–1279, 10.4161/cbt.29691. 24971465 PMC4128869

[49] J. Kang , H. Jung , and H. Kim , “Prognostic Value of Cyclin B1 and Cyclin B2 Expression in Breast Cancer: A Systematic Review and Updated Meta‐analysis,” Medicine 103, no. 3 (2024): e37016, 10.1097/md.0000000000037016. 38241547 PMC10798710

[50] I. Androic , A. Krämer , R. Yan , et al., “Targeting Cyclin B1 Inhibits Proliferation and Sensitizes Breast Cancer Cells to Taxol,” BMC Cancer 8 (2008): 391, 10.1186/1471-2407-8-391. 19113992 PMC2639606

