# Supplementary material for: Identification of a Circular RNA as a Potential Diagnostic and Prognostic Biomarker in Breast Cancer Through Integrated Bioinformatic and Experimental Analyses
Source: Anal Sci Adv. 2026 Jul 28;7(2):e70098. doi: 10.1002/ansa.70098 (PMC13412398; doi:10.1002/ansa.70098)
Supplement: Supplementary file 1 — Supporting File: ansa70098‐sup‐0001‐SuppMat.pdf. [file ANSA-7-e70098-s001.pdf]

R scripts used for differential expression analysis and data processing:

GEO datasets:

```
#Calling necessary packages

require(BiocManager)
require(limma)
require(GEOquery)
require(data.table)
require(Biobase)
require(ggplot2)
require(gplots)
require(sva)
require(umap)
require(pheatmap)

WD<-getwd()
setwd(WD)

###################### GSE101123_series_matrix GPL19978 ######################

gset1<- getGEO("GSE101123",GSEMatrix = T,getGPL = T,destdir = "")
gset1
class(gset1)
gset1<-gset1[[1]]
dim(gset1)

# View(fData(gset1)) ## View the gene annotation
# View(exprs(gset1)) ## View the expression data
```

```
ex1<-exprs(gset1)
ex1<-as.data.frame(ex1)
max(ex1)
min(ex1)

annot1_GPL19978<-fData(gset1)

###################### GSE165884_series_matrix GPL21825 ########################

gset2<- getGEO("GSE165884",GSEMatrix = T,getGPL = T,destdir = WD)
gset2
class(gset2)
gset2<-gset2[[1]]
dim(gset2)

# View(fData(gset2)) ## View the gene annotation
# View(exprs(gset2)) ## View the expression data

ex2<-exprs(gset2)
ex2<-as.data.frame(ex2)
max(ex2)
min(ex2)

annot2_GPL21825<-fData(gset2)

####################### GSE182471_series_matrix GPL21825 #######################

gset3<- getGEO("GSE182471",GSEMatrix = T,getGPL = F,destdir = WD)
gset3
class(gset3)
```

```
gset3<-gset3[[1]]
dim(gset3)

# View(exprs(gset2))

ex3<-exprs(gset3)
ex3<-as.data.frame(ex3)
max(ex3)
min(ex3)

# All data are in log scale and doesn't need to normalization

################################ Annotation match ###############################
annot1_GPL19978 <- annot1_GPL19978[,c("ID","circRNA")]
annot2_GPL21825 <- annot2_GPL21825[,c("ID","circRNA")]

rownames(ex1) <- annot1_GPL19978[as.character(rownames(ex1)), "circRNA"]
rownames(ex2) <- annot2_GPL21825[as.character(rownames(ex2)), "circRNA"]
rownames(ex3)<- annot2_GPL21825[as.character(rownames(ex3)), "circRNA"]

ex1$Row.names<-rownames(ex1)
ex1<-ex1[,c(12,1:11)]
ex2$Row.names<-rownames(ex2)
ex2<-ex2[,c(9,1:8)]
ex3$Row.names<-rownames(ex3)
ex3<-ex3[,c(11,1:10)]

datamerge1 <- merge(ex1,ex2,by="Row.names")
datamerge <- merge(datamerge1,ex3,by="Row.names")
rownames(datamerge)<-datamerge$Row.names
```

```
datamerge<-datamerge[,-1]

par(mar=c(8,2,1,1))
pdf("1-boxplot_before_batcheffect.pdf", width = 9, height = 9, bg = "white",)
boxplot(datamerge, outline=F, Logic=T, varwidth=T, notch=T, col="#FFB90F",
        ylab="Log2 Transformed Expression", las=2,
        names.arg=colnames(datamerge), cex.axis=0.7)
dev.off()

ex1<-ex1[,-1]
ex2<-ex2[,-1]
ex3<-ex3[,-1]

batch<-factor(c(rep(1,ncol(ex1)),rep(2,ncol(ex2)),rep(3,ncol(ex3))))

allm <- datamerge
pc<-prcomp(allm)
pcr<-data.frame(pc$r[,1:3],batch)
pdf("2-PC1_before_batcheffect.pdf", width = 9, height = 9, bg = "white",)
ggplot(pcr,aes(PC1,PC2,color=batch))+geom_point()+theme_bw()
dev.off()

allc<-ComBat(datamerge,batch)
par(mar=c(8,2,1,1))
pdf("3-boxplot_after_batcheffect.pdf", width = 9, height = 9, bg = "white",)
boxplot(allc,outline=F,Logic=T,varwidth=T,notch=T,col="#FFB90F",
        ylab="Log2 Transformed Expression",las=2,
        names.arg=colnames(datamerge), cex.axis=0.7)
dev.off()
```

```
pc<-prcomp(allc)
pcr<-data.frame(pc$r[,1:3],batch)
pdf("4-PC1_after_batcheffect.pdf", width = 9, height = 9, bg = "white",)
ggplot(pcr,aes(PC1,PC2,color=batch))+geom_point()+theme_bw()
dev.off()

################################ defining groups ###############################
# View the sample information
# View(pData(gset1))
# View(pData(gset2))
# View(pData(gset3))

gr<-c(rep("Cancer",8),rep("Control",3),rep("Cancer",4),rep("Control",4),
   rep("Control",5),rep("Cancer",5))
length(gr)

################################## Correlation ################################

pheatmap(cor(allc))
pheatmap(cor(allc),labels_row = gr,labels_col = gr)

######################## Design matrix for limma package #######################
gr<-factor(gr)
design <- model.matrix(~0 + gr)
colnames(design) <- levels(gr)

fit <- lmFit(allc, design)

# set up contrasts of interest and recalculate model coefficients
```

```
cont.matrix <- makeContrasts(Cancer-Control, levels=design)
fit2 <- contrasts.fit(fit, cont.matrix)

# compute statistics and table of top significant genes
fit2 <- eBayes(fit2, 0.01)
tT <- topTable(fit2, adjust="fdr", sort.by="B", number=Inf)
tT <- subset(tT, select=c("adj.P.Val","P.Value","logFC"))
write.csv(tT, "5-tT1.csv", row.names=T, sep="\t")

DEcircRNA<-subset(tT,adj.P.Val < 0.05 & abs(logFC) > 1)

url <- "https://raw.githubusercontent.com/pejmanmorovat/Annotation/main/Annotation.csv"
destfile <- "Annotation.csv"
download.file(url, destfile)
annot<-read.csv("Annotation.csv")
rownames(annot)<-annot$circRNA
DEcirc<-merge(annot,DEcircRNA,by=0)
write.csv(DEcirc,"6-40DEcircRNA.csv",row.names=F, sep="\t")

DEcirc_UP<-DEcirc[which(DEcirc$logFC >1 & DEcirc$adj.P.Val< 0.05),]
dim(DEcirc)
write.csv(DEcirc_UP,"7-Upregulated_DEcircRNAs.csv",row.names = F)
DEcirc_Down<-DEcirc[which(DEcirc$logFC < -1 & DEcirc$adj.P.Val< 0.05),]
dim(DEcirc_Down)
write.csv(DEcirc_Down,"8-Downregulated_DEcircRNAs.csv",row.names = F)

################################ Volcano plot ##############################

library(EnhancedVolcano)
library(extrafont)
```

```
library(gridExtra)

pc<-tT

keyvals <- rep("black", nrow(pc))
names(keyvals) <- rep("No Significance", nrow(pc))

keyvals[which(pc$logFC > 1 & pc$adj.P.Val < 0.05)] <- "red"
names(keyvals)[which(pc$logFC > 1 & pc$adj.P.Val <0.05)] <- "Upregulate"

keyvals[which(pc$logFC < -1 & pc$adj.P.Val < 0.05)] <- "green"
names(keyvals)[which(pc$logFC < -1 & pc$adj.P.Val <0.05)] <- "Downregulate"

pdf("9-volcano_toptable.pdf", width = 12, height = 9, bg = "white",)
EnhancedVolcano(pc,lab = rownames(pc),x = 'logFC',y = 'adj.P.Val',pCutoff = 0.05,
        FCcutoff = 1,cutoffLineType = 'twodash',cutoffLineWidth = 0.9,cutoffLineCol =
"orange",
        pointSize = 2,labSize = 6.0,colAlpha = 1,
        legendPosition = 'top',legendLabSize = 16,legendIconSize = 5.0,
        subtitle = "(a)",titleLabSize = 10,colCustom = keyvals,
        selectLab = rownames(pc)[which(names(keyvals) %in% c("High", "Low"))],
        title = "", border = "full",borderWidth = 1.0,borderColour = "black",
        gridlines.major = FALSE,gridlines.minor = FALSE,xlim = c(-2.5,2.5),
        ylim = c(0, max(-log10(pc[[1]]), na.rm = TRUE) + 2),
        ylab = bquote(~-Log[10] ~ italic(adj.P.Val)))
dev.off()

################################## Heatmap ###################################

library(pheatmap)
```

```r
library(viridis)
library(gplots)

DEC<-as.character(rownames(DEcircRNA))

DEheatmap <- allc[DEC,]
col <-as.data.frame(gr)
rownames(col) <- colnames(DEheatmap)
colnames(col)[1] <- "Groupe"

col$Groupe <- factor(col$Groupe, levels = c("Cancer", "Control"))
my_colour = list(Groupe = c(Cancer = "darkgoldenrod2", Control = "#5977ff"),
                random = c(random1 = "#82ed82", random2 = "#9e82ed"),
                cluster = c(cluster1 = "#e89829", cluster2 = "#cc4ee0"))

pheatmap(DEheatmap,border_color = NA,color = greenred(256),
         display_numbers = F,number_color = "white",annotation_col = col,
         annotation_colors= my_colour)

#################################### boxplot ##################################
library(grid)
library(ggplot2)
library(gridExtra)
library(tidyverse)
library(ggthemes)
library(reshape)

full_inf<-merge(tT,annot,by=0)

circ1<-"hsa_circ_0000231"
```

```
circ2<-"hsa_circ_0011385"

################################ boxplot of circRNA 1 #############################
number1<-which(full_inf$Alias==circ1)
name1<-full_inf[number1,]
circRNA_name1<-name1[,"circRNA"]



hsa_circ1<- data.frame(allc[circRNA_name1,])
hsa_circ1$type <- gr
colnames(hsa_circ1) <- c("value","type")

theme_complete_bw <- function(base_size = 24, base_family = "")
{
  theme_grey(base_size = base_size, base_family = base_family) %+replace%
    theme(
      axis.line =         element_blank(),
      axis.text.x =       element_text(size = base_size * 0.8 , lineheight = 0.9, colour = "black", vjust
= 0.5),
      axis.text.y =       element_text(size = base_size * 0.8, lineheight = 0.9, colour = "black", hjust
= 0.5),
      axis.ticks =        element_line(colour = "black"),
      axis.title.x =      element_text(size = base_size, vjust = 0),
      axis.title.y =      element_text(size = base_size, angle = 90, vjust = 2),
      axis.ticks.length = unit(0.15, "cm"),
      axis.ticks.margin = unit(0.1, "cm"),

      legend.background = element_rect(colour=NA),
      legend.key =        element_rect(fill =NA, colour = "black", size = 0.25),
      legend.key.size =   unit(1.5, "lines"),
```

```
      legend.text =       element_text(size = base_size * 0.7),
      legend.title =      element_text(size = base_size * 0.8),
      legend.position =   "top",

      panel.background = element_rect(fill = "white", colour = NA),
      panel.border =     element_rect(fill = NA, colour = "black", size=2),
      panel.grid.major = element_line(colour = NA, size = 0.2), #"grey"
      panel.grid.minor = element_line(colour = NA, size = 0.5), #"grey"
      panel.margin =     unit(0.25, "lines"),

      strip.background = element_rect(fill = NA, colour = NA),
      strip.text.x =     element_text(colour = "black", size = base_size * 0.8),
      strip.text.y =     element_text(colour = "black", size = base_size * 0.8, angle = +90),

      plot.background =  element_rect(colour = NA, fill = "white"),
      plot.title =       element_text(size = base_size*.8),
      plot.margin =      unit(c(1, 1, .5, .5), "lines"))
}

pdf("10-boxplot_circ_0000231.pdf",width = 10,height = 10,bg = "white")
ggplot(hsa_circ1,aes(x=type,y=value,fill=type))+geom_boxplot(outlier.shape = NA)+
  xlab("Sample")+ylab("Expression of DEC")+ theme_complete_bw()+scale_fill_few()
dev.off()

############################## boxplot of circRNA 2 ##############################

number2<-which(full_inf$Alias==circ2)
name2<-full_inf[number2,]
circRNA_name2<-name2[,"circRNA"]
```

```
hsa_circ2<- data.frame(allc[circRNA_name2,])
hsa_circ2$type <- gr
colnames(hsa_circ2) <- c("value","type")

theme_complete_bw <- function(base_size = 24, base_family = "")
{
  theme_grey(base_size = base_size, base_family = base_family) %+replace%
    theme(
      axis.line =         element_blank(),
      axis.text.x =       element_text(size = base_size * 0.8 , lineheight = 0.9, colour = "black", vjust = 0.5),
      axis.text.y =       element_text(size = base_size * 0.8, lineheight = 0.9, colour = "black", hjust = 0.5),
      axis.ticks =        element_line(colour = "black"),
      axis.title.x =      element_text(size = base_size, vjust = 0),
      axis.title.y =      element_text(size = base_size, angle = 90, vjust = 2),
      axis.ticks.length = unit(0.15, "cm"),
      axis.ticks.margin = unit(0.1, "cm"),

      legend.background = element_rect(colour=NA),
      legend.key =        element_rect(fill =NA, colour = "black", size = 0.25),
      legend.key.size =   unit(1.5, "lines"),
      legend.text =       element_text(size = base_size * 0.7),
      legend.title =      element_text(size = base_size * 0.8),
      legend.position =   "top",

      panel.background = element_rect(fill = "white", colour = NA),
      panel.border =     element_rect(fill = NA, colour = "black", size=2),
      panel.grid.major = element_line(colour = NA, size = 0.2), #"grey"
      panel.grid.minor = element_line(colour = NA, size = 0.5), #"grey"
```

```
    panel.margin =     unit(0.25, "lines"),

    strip.background = element_rect(fill = NA, colour = NA),
    strip.text.x =     element_text(colour = "black", size = base_size * 0.8),
    strip.text.y =     element_text(colour = "black", size = base_size * 0.8, angle = +90),

    plot.background =  element_rect(colour = NA, fill = "white"),
    plot.title =       element_text(size = base_size*.8),
    plot.margin =      unit(c(1, 1, .5, .5), "lines"))
}

pdf("11-boxplot_circ_0011385.pdf",width = 10,height = 10,bg = "white")
ggplot(hsa_circ2,aes(x=type,y=value,fill=type))+geom_boxplot(outlier.shape = NA)+
  xlab("Sample")+ylab("Expression of DEC")+ theme_complete_bw()+scale_fill_few()
dev.off()
```

TCGA-BRCA datasets:

```
#Calling necessary packages

library(GDCRNATools)

library(pathview)

library(edgeR)

library(limma)

library(DESeq2)

library(data.table)

library(ggplot2)

library(reshape2)
```

```
library(plyr)
library(survival)
library(survminer)
library(dplyr)
library(RColorBrewer)
options(digits = 3)
options(stringsAsFactors = F)

project <- 'TCGA-BRCA'
rnadir <- paste(project, 'RNAseq', sep='/')
mirdir <- paste(project, 'miRNAs', sep='/')
clinicaldir <- paste(project, 'Clinical', sep='/')

# gdcRNADownload(project.id     = 'TCGA-BRCA',
#                data.type      = 'RNAseq',
#                write.manifest = FALSE,
#                 method         = 'gdc-client',
#                 directory      = rnadir)

# gdcRNADownload(project.id     = 'TCGA-BRCA',
#                data.type      = 'miRNAs',
#                write.manifest = FALSE,
#                method         = 'gdc-client',
```

```
#           directory     = mirdir)



# gdcClinicalDownload(project.id     = 'TCGA-BRCA',
#                 write.manifest = FALSE,
#                 method         = 'gdc-client',
#                 directory      = clinicaldir)

############################## Parse RNAseq metadata ############################

# metaMatrix.RNA <- gdcParseMetadata(project.id = 'TCGA-BRCA',
#                               data.type  = 'RNAseq',
#                               write.meta = FALSE)

# saveRDS(object = metaMatrix.RNA,file = "metaMatrixRNA.RDS",compress = FALSE)


metaMatrix.RNA = readRDS(file = "metaMatrixRNA.RDS")
table(metaMatrix.RNA$sample_type)
metaMatrix.RNA <- gdcFilterDuplicate(metaMatrix.RNA)
metaMatrix.RNA <- gdcFilterSampleType(metaMatrix.RNA)
table(metaMatrix.RNA$sample_type)
```

```
############################## Parse miRNAs metadata ############################

# metaMatrix.MIR <- gdcParseMetadata(project.id = 'TCGA-BRCA',
#                                   data.type  = 'miRNAs',
#                                   write.meta = FALSE)

# saveRDS(object = metaMatrix.MIR,file = "metaMatrixMIR.RDS",compress = FALSE)


metaMatrix.MIR = readRDS(file = "metaMatrixMIR.RDS")
table(metaMatrix.MIR$sample_type)
metaMatrix.MIR <- gdcFilterDuplicate(metaMatrix.MIR)
metaMatrix.MIR <- gdcFilterSampleType(metaMatrix.MIR)
table(metaMatrix.MIR$sample_type)


############################### Merge RNAseq data #############################

# rnaCounts <- gdcRNAMerge(metadata  = metaMatrix.RNA,
#                          path      = rnadir, # the folder in which the data stored
#                          organized = FALSE, # if the data are in separate folders
#                          data.type = 'RNAseq')
rnaCounts<-read.table("rnacounts.txt")
```

```r
############################## Merge miRNAs data #############################

# mirCounts <- gdcRNAMerge(metadata  = metaMatrix.MIR,
#                          path      = mirdir, # the folder in which the data stored
#                          organized = FALSE, # if the data are in separate folders
#                          data.type = 'miRNAs')
mirCounts<-read.table("mirCount.txt")

################################ Merge clinical data ################################

# clinicalDa <- gdcClinicalMerge(path = clinicaldir, key.info = TRUE)

rnaExpr <- gdcVoomNormalization(counts = rnaCounts, filter = FALSE)
mirExpr <- gdcVoomNormalization(counts = mirCounts, filter = FALSE)

# DEGAll <- gdcDEAnalysis(counts     = rnaCounts,
#                         group      = metaMatrix.RNA$sample_type,
#                         comparison = 'PrimaryTumor-SolidTissueNormal',
#                         method     = 'DESeq2')

DEGAll<-read.table("results/DEGAll.txt")
```

```
################################## All DEGs #####################################


# deALL <- gdcDEReport(deg = DEGAll, gene.type = 'all')

deALL<-read.table("results/deALL.txt")

table(deALL$group)


############################## DE protein coding genes ############################

# dePC <- gdcDEReport(deg = DEGAll, gene.type = 'protein_coding')

dePC<-read.table("results/dePC.txt")


#################################### plot ########################################


gdcBarPlot(deg = deALL, angle = 45, data.type = 'RNAseq')

degName = rownames(deALL)

# gdcHeatmap(deg.id = degName, metadata = metaMatrix.RNA, rna.expr = rnaExpr)


################################## DEGmir #######################################


DEGmir <- gdcDEAnalysis(counts     = mirCounts,

                        group      = metaMatrix.MIR$sample_type,

                        comparison = 'PrimaryTumor-SolidTissueNormal',

                        method     = 'DESeq2')
```

```
demirALL <- gdcDEReport(deg = DEGmir,fc=3)

mirup <- subset(demirALL,logFC > 1)
mirdown <- subset(demirALL,logFC < 1)

############################## Volcano plot miRNAs ############################

library(EnhancedVolcano)
library(extrafont)
library(gridExtra)

pc<-DEGmir
pc<-pc[,-c(1,3,4)]
names(pc)<-c("logFC","PValue","adj.P.Val")

keyvals <- rep("black", nrow(pc))
names(keyvals) <- rep("No Significance", nrow(pc))

keyvals[which(pc$logFC > 1 & pc$adj.P.Val < 0.05)] <- "red"
names(keyvals)[which(pc$logFC > 1 & pc$adj.P.Val <0.05)] <- "Upregulate"
```

```
keyvals[which(pc$logFC < -1 & pc$adj.P.Val < 0.05)] <- "blue"

names(keyvals)[which(pc$logFC < -1 & pc$adj.P.Val <0.05)] <- "Downregulate"


EnhancedVolcano(pc,lab = rownames(pc),x = 'logFC',y = 'adj.P.Val',pCutoff = 0.05,
        FCcutoff = 1,cutoffLineType = 'twodash',cutoffLineWidth = 0.9,cutoffLineCol =
"orange",
        pointSize = 2,labSize = 6.0,colAlpha = 1,
        legendPosition = 'top',legendLabSize = 16,legendIconSize = 5.0,
        subtitle = "(a)",titleLabSize = 10,colCustom = keyvals,
        selectLab = rownames(pc)[which(names(keyvals) %in% c("High", "Low"))],
        title = "", border = "full",borderWidth = 1.0,borderColour = "black",
        gridlines.major = FALSE,gridlines.minor = FALSE,xlim = c(-4,4),
        ylab = bquote(~-Log[10] ~ italic(adj.P.Val)))


############################### Volcano plot mRNAs ############################


pc<-DEGAll

pc<-pc[,c(4,7,8)]

names(pc)<-c("logFC","PValue","adj.P.Val")


keyvals <- rep("black", nrow(pc))

names(keyvals) <- rep("No Significance", nrow(pc))
```

```
keyvals[which(pc$logFC > 1 & pc$adj.P.Val < 0.05)] <- "red"

names(keyvals)[which(pc$logFC > 1 & pc$adj.P.Val <0.05)] <- "Upregulate"


keyvals[which(pc$logFC < -1 & pc$adj.P.Val < 0.05)] <- "blue"

names(keyvals)[which(pc$logFC < -1 & pc$adj.P.Val <0.05)] <- "Downregulate"


EnhancedVolcano(pc,lab = rownames(pc),x = 'logFC',y = 'adj.P.Val',pCutoff = 0.05,

        FCcutoff = 1,cutoffLineType = 'twodash',cutoffLineWidth = 0.9,cutoffLineCol = 
"orange",

        pointSize = 2,labSize = 6.0,colAlpha = 1,

        legendPosition = 'top',legendLabSize = 16,legendIconSize = 5.0,

        subtitle = "(a)",titleLabSize = 10,colCustom = keyvals,

        selectLab = rownames(pc)[which(names(keyvals) %in% c("High", "Low"))],

        title = "", border = "full",borderWidth = 1.0,borderColour = "black",

        gridlines.major = FALSE,gridlines.minor = FALSE,xlim = c(-4,4),

        ylab = bquote(~-Log[10] ~ italic(adj.P.Val)))


########################## Expression mRNAs Boxplot ############################


library(RColorBrewer)

library(viridis)

library(extrafont)
```

```
library(ggthemes)


theme_complete_bw <- function(base_size = 24, base_family = "")
{
  theme_grey(base_size = base_size, base_family = base_family) %+replace%
    theme(
      axis.line =         element_blank(),
      axis.text.x =       element_text(size = base_size * 0.8 , lineheight = 0.9, colour = "black", vjust
= 0.5),
      axis.text.y =       element_text(size = base_size * 0.8, lineheight = 0.9, colour = "black", hjust
= 0.5),
      axis.ticks =        element_line(colour = "black"),
      axis.title.x =      element_text(size = base_size, vjust = 0),
      axis.title.y =      element_text(size = base_size, angle = 90, vjust = 2),
      axis.ticks.length = unit(0.15, "cm"),
      axis.ticks.margin = unit(0.1, "cm"),

      legend.background = element_rect(colour=NA),
      legend.key =        element_rect(fill =NA, colour = "black", size = 0.25),
      legend.key.size =   unit(1.5, "lines"),
      legend.text =       element_text(size = base_size * 0.7),
      legend.title =      element_text(size = base_size * 0.8),
      legend.position =   "top",
```

```
    panel.background = element_rect(fill = "white", colour = NA),
    panel.border =     element_rect(fill = NA, colour = "black", size=2),
    panel.grid.major = element_line(colour = NA, size = 0.2), #"grey"
    panel.grid.minor = element_line(colour = NA, size = 0.5), #"grey"
    panel.margin =     unit(0.25, "lines"),

    strip.background = element_rect(fill = NA, colour = NA),
    strip.text.x =     element_text(colour = "black", size = base_size * 0.8),
    strip.text.y =     element_text(colour = "black", size = base_size * 0.8, angle = +90),

    plot.background =  element_rect(colour = NA, fill = "white"),
    plot.title =       element_text(size = base_size*.8),
    plot.margin =      unit(c(1, 1, .5, .5), "lines"))
}

CCNB1<-"ENSG00000134057"
FOXM1<-"ENSG00000111206"

aa<- data.frame(rnaExpr[CCNB1,])
aa$type <- metaMatrix.RNA$sample_type
colnames(aa) <- c("value","type")
```

```
ggplot(aa,aes(x=type,y=value,fill=type))+geom_boxplot(outlier.shape = NA)+
  xlab("Sample")+ylab("Expression level (CCNB1)")+
  theme_complete_bw()+scale_fill_few()

bb<- data.frame(rnaExpr[FOXM1,])
bb$type <- metaMatrix.RNA$sample_type
colnames(bb) <- c("value","type")

ggplot(bb,aes(x=type,y=value,fill=type))+geom_boxplot(outlier.shape = NA)+
  xlab("Sample")+ylab("Expression level (FOXM1)")+
  theme_complete_bw()+scale_fill_few()

######################## Expression of miRNAs Boxplot ##########################

cc<- data.frame(mirExpr["hsa-miR-5683",])
cc$type <- metaMatrix.MIR$sample_type
colnames(cc) <- c("value","type")

ggplot(cc,aes(x=type,y=value,fill=type))+geom_boxplot(outlier.shape = NA)+
  xlab("Sample")+ylab("Expression level (hsa-miR-5683)")+
  theme_complete_bw()+scale_fill_few()

dd<- data.frame(mirExpr["hsa-miR-204-5p",])
```

```r
dd$type <- metaMatrix.MIR$sample_type

colnames(dd) <- c("value","type")


ggplot(dd,aes(x=type,y=value,fill=type))+geom_boxplot(outlier.shape = NA)+

  xlab("Sample")+ylab("Expression level (hsa-miR-204-5p)")+

  theme_complete_bw()+scale_fill_few()
```
